# Mesoscale activity drives the habitat suitability of yellowfin tuna in the Gulf of Mexico

**DOI:** 10.1038/s41598-024-58613-7

**Published:** 2024-04-08

**Authors:** Zurisaday Ramírez-Mendoza, Oscar Sosa-Nishizaki, Mario A. Pardo, Sharon Z. Herzka, R. J. David Wells, Jay R. Rooker, Brett J. Falterman, Michel J. Dreyfus-León

**Affiliations:** 1 Fisheries Ecology Laboratory, Departamento de Oceanografía Biológica. Centro de Investigación Científica y de Educación Superior de Ensenada (CICESE), 22860 Ensenada, Baja California Mexico; 2 Marine Macroecology Laboratory, Unidad la Paz, CICESE-Consejo Nacional de Humanidades, Ciencias y Tecnologías (CONAHCYT), 23050 La Paz, Baja California Sur Mexico; 3 Department of Marine Science, Marine Science Institute, University of Texas at Austin, Port Aransas, TX 78373 USA; 4 Department of Marine Biology, Texas A&M University at Galveston, Galveston, TX 77553 USA; 5 Department of Ecology and Conservation Biology, Texas A&M University, College Station, TX 77843 USA; 6 Fisheries Research Support LLC., Mandeville, LA 70448 USA; 7 Programa Nacional de Aprovechamiento del Atún y Protección del Delfín, CICESE, 22860 Ensenada, Baja California Mexico

**Keywords:** Pelagic fisheries, Bayesian models, INLA, Eddies, Habitat suitability, Ecology, Climate sciences, Ecology, Environmental sciences, Ocean sciences

## Abstract

Yellowfin tuna, *Thunnus albacares*, represents an important component of commercial and recreational fisheries in the Gulf of Mexico (GoM). We investigated the influence of environmental conditions on the spatiotemporal distribution of yellowfin tuna using fisheries’ catch data spanning 2012–2019 within Mexican waters. We implemented hierarchical Bayesian regression models with spatial and temporal random effects and fixed effects of several environmental covariates to predict habitat suitability (HS) for the species. The best model included spatial and interannual anomalies of the absolute dynamic topography of the ocean surface (ADT_SA_ and ADT_IA_, respectively), bottom depth, and a seasonal cyclical random effect. High catches occurred mainly towards anticyclonic features at bottom depths > 1000 m. The spatial extent of HS was higher in years with positive ADT_IA_, which implies more anticyclonic activity. The highest values of HS (> 0.7) generally occurred at positive ADT_SA_ in oceanic waters of the central and northern GoM. However, high HS values (> 0.6) were observed in the southern GoM, in waters with cyclonic activity during summer. Our results highlight the importance of mesoscale features for the spatiotemporal distribution of yellowfin tunas and could help to develop dynamic fisheries management strategies in Mexico and the U.S. for this valuable resource.

## Introduction

Understanding the relationship between fish populations and their dynamic environment is fundamental for pelagic fish stock assessment, ecosystem management, and conservation^1,2^. The distribution of highly migratory large pelagic fishes (e.g., tunas, sharks, or billfishes) is related to specific environmental conditions that fulfill each species’ requirements, which may change throughout their life history^3,4^. These include predation^5^, larval survival^6^, reproduction^4^, and movements^7^.

**Figure 1:**
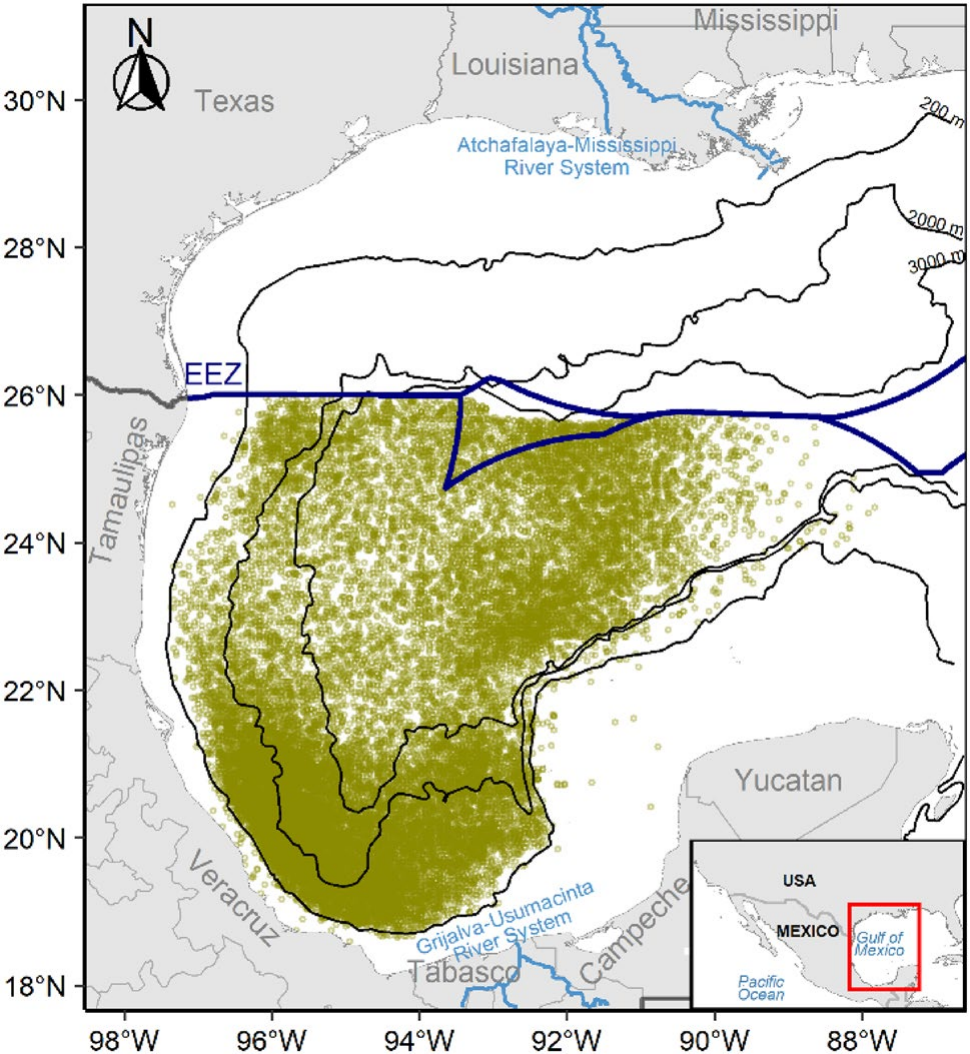
Locations of yellowfin tuna fishery longline sets deployed by the Mexican fleet (yellow circles; n = 26,515) in the Gulf of Mexico. The dark blue line shows the boundary between Mexican and U.S. Exclusive Economic Zones (EEZ). Black lines are the smoothed 200-, 2000-, and 3000-m isobaths. The map was created with R’s package “ggplot2” (https://ggplot2.tidyverse.org/), using the coastlines and political boundaries from the Global Self-consistent, Hierarchical, High-resolution Geography Database (http://www.soest.hawaii.edu/pwessel/gshhg/), and the topography data from the Scripps Institution of Oceanography’s Satellite Geodesy (https://topex.ucsd.edu/marine_topo/).

Yellowfin tuna, *Thunnus albacares*, is a large pelagic fish inhabiting all ocean’s tropical and subtropical waters^8^. This apex predator plays a vital role in regulating energy flows between different trophic levels^9^ and supports highly valuable fisheries worldwide. It accounts for ~ 25% of the annual global tuna catch, making it the second most fished tuna species globally^10^. In the Atlantic Ocean, it is managed as a single panmictic (i.e., mixed) stock, and according to the most recent assessment, it is not overfished^11^. In the Gulf of Mexico (GoM), this species represents an important component of the United States and Mexican commercial and recreational fisheries, mainly during the summer months, with more than 2000-t landings per year by both fisheries^12,13^. In the southern GoM, within the Mexican Exclusive Economic Zone (Fig. 1), yellowfin tuna is the main target species of the Mexican commercial longline fleet^12^.

The distribution and movement patterns of tunas are influenced by factors such as temperature and dissolved oxygen^3^, spawning activity, and prey availability^14,15^. Yellowfin tuna prefer warm waters (> 20 °C)^15,16^, associated with highly productive areas influenced by temperature fronts and mesoscale eddies^7,17^. Vertically, the species prefers shallow warm waters of the mixed layer above the thermocline (18–31°), although occasionally, they perform short deep dives (> 300 m)^16,18^.

In the northern GoM, yellowfin tuna exhibits some degree of residency^15,17^, although part of the population performs large-scale movements between the northern and southern GoM related to feeding and seasonal spawning^12,19–21^. Year-round catches of yellowfin tuna by the Mexican longline fleet confirm the central and southern GoM as important core habitats^12^. In the GoM, the species seems to be less sensitive to environmental variability than other tunas^13^, besides a stable association to warm (28–30 °C) and oligotrophic waters, as well as to positive sea level anomalies associated to anticyclonic circulation^19^. Nevertheless, the spatiotemporal distribution of yellowfin tuna and its main environmental drivers in the GoM remain unclear.

The GoM is a large, semi-enclosed basin connected to the Atlantic Ocean and the Caribbean Sea. This region is influenced by mesoscale circulation that modulates the productivity in the area (Fig. 1, p. 1473 in^22^). Its circulation in oceanic waters is dominated by the Loop Current, which flows northward through the Yucatan Channel and makes an anticyclonic turn before exiting through the Florida Straits^23^. The Loop Current shows different degrees of intrusion into the GoM and generates cyclonic and anticyclonic eddies^24^. In contrast, in the southern GoM, the large Bay of Campeche (south of 22° N) exhibits a semi-permanent cyclonic eddy^25^ and seasonal cross-shelf transport^26,27^ that supports high biological productivity, leading to high prey biomass for top predators. It is also influenced by the freshwater discharge of the Grijalva-Usumacinta River System and regional upwelling that also enhances productivity^28^.

Species distribution models of large pelagic fishes have been extensively used to predict suitable areas^29^, identify hotspots^1,30^, and assess the potential impacts of climate change^31^ and of anthropogenic events such as oil spills^30,32^ or fishing mortality^13,19^. We implemented mixed-effects Bayesian spatial models with an integrated nested Laplace approximation (INLA)^33^, which has demonstrated to be a great alternative for modeling species distributions and environmental preferences^1,34^, giving results in terms of posterior probability distributions instead of fixed values, and incorporating several types of random effects of both spatial and temporal nature, with reduced computational times^35,36^.

This study aimed to predict the spatiotemporal distribution of yellowfin tuna in the GoM as a function of environmental conditions. We tested several model structures with combinations of environmental covariates, based on the hypothesis that this highly migratory tuna shows affinities for ocean fronts and eddies, and it uses specific regions of high primary and secondary production and, therefore, higher prey availability. We also expected variations of habitat suitability at seasonal, interannual, and long-term scales associated with the species' life history and the GOM’s environmental dynamics. We used the best model results to produce monthly spatial predictions of the species’ habitat suitability for contrasting years and for a 10-year climatology.

## Results

Exploratory analysis of the fishery data showed that the Mexican longline fishery is focused on adult yellowfin tuna (Supplementary Fig. S4). The sex ratio showed that more males were caught from 2012 to 2019 (Supplementary Table S1). The highest mean CPUE occurred in December (18.47 ± 4.59) and May (16.09 ± 2.85), and the lowest in February (10.55 ± 1.95) and September (9.91 ± 1.95) (Supplementary Fig. S5). Interannual variability showed that the highest CPUE occurred in 2016 (17.66 ± 3.22), while the lowest was in 2019 (11.41 ± 2.53) (Supplementary Fig. S6).

The number of yellowfin tuna caught in a single longline set varied between 0 and 105. Only 4% (952) of the sets had zero catches, and there was no need to use zero-inflated likelihoods (Supplementary Fig. S7). None of the predictors showed outliers (Supplementary Fig. S8). Variance inflation factors (VIF > 3) and correlation matrix values (|r|> 0.6) indicated collinearity between CHL and CHL_SA_, and ADT and ADT_SA_. Those pairs were not included in the same model structure (Supplementary Files: Fig. S9 and Table S2). For all competing models, the negative binomial likelihood represented better the response variable rather than the Poisson distribution. We compared the best negative binomial model (Table 1) with the best Poisson model and found that the negative binomial (WAIC = 157,513.10) had a better fit than the respective Poisson model (WAIC = 207,357.60, ΔWAIC = 49,844.5).

**Table 1:** The best five models of yellowfin tuna catches (*µ*_*y*_) as a function of habitat, sorted by ascending Watanabe-Akaike Information Criterion (WAIC).

Model structure	WAIC	ΔWAIC
${\mu }_{{y}_{i}}\sim AD{T}_{SAi}+AD{T}_{SAi}^{2}+AD{T}_{SAi}^{3}+B{D}_{i}+B{D}_{i}^{2}+B{D}_{i}^{3}+AD{T}_{IAi}+AD{T}_{IAi}^{2}+AD{T}_{IAi}^{3}+{W}_{i}+\gamma \cdot {M}_{i}$	157,513.10	0
${\mu }_{{y}_{i}}\sim AD{T}_{SAi}+AD{T}_{SAi}^{2}+B{D}_{i}+B{D}_{i}^{2}+AD{T}_{IAi}+AD{T}_{IAi}^{2}+{W}_{i}+\gamma \cdot {M}_{i}$	157,516.00	2.9
${\mu }_{{y}_{i}}\sim AD{T}_{SAi}+AD{T}_{SAi}^{2}+B{D}_{i}+B{D}_{i}^{2}+AD{T}_{IAi}+{W}_{i}+\gamma \cdot {M}_{i}$	157,518.20	5.1
${\mu }_{{y}_{i}}\sim AD{T}_{SAi}+AD{T}_{SAi}^{2}+AD{T}_{SAi}^{3}+B{D}_{i}+B{D}_{i}^{2}+B{D}_{i}^{3}+AD{T}_{IAi}+AD{T}_{IAi}^{2}+{W}_{i}+\gamma \cdot {M}_{i}$	157,523.10	10.0
${\mu }_{{y}_{i}}\sim AD{T}_{SAi}+AD{T}_{SAi}^{2}+AD{T}_{SAi}^{3}+B{D}_{i}+B{D}_{i}^{2}+B{D}_{i}^{3}+AD{T}_{IAi}+{W}_{i}+\gamma \cdot {M}_{i}$	157,525.40	12.3

Covariates include absolute dynamic topography spatial anomalies (*ADT*_*SA*_), absolute dynamic topography interannual anomalies (*ADT*_*IA*_), sea surface temperature spatial anomalies (*SST*_*SA*_), sea surface temperature interannual anomalies (*SST*_*IA*_), and bottom depth (*BD*), as well as a spatial random effect (*W*) and a seasonal random effect (*γ*) of the month of the year (*M*).

**Figure 2:**
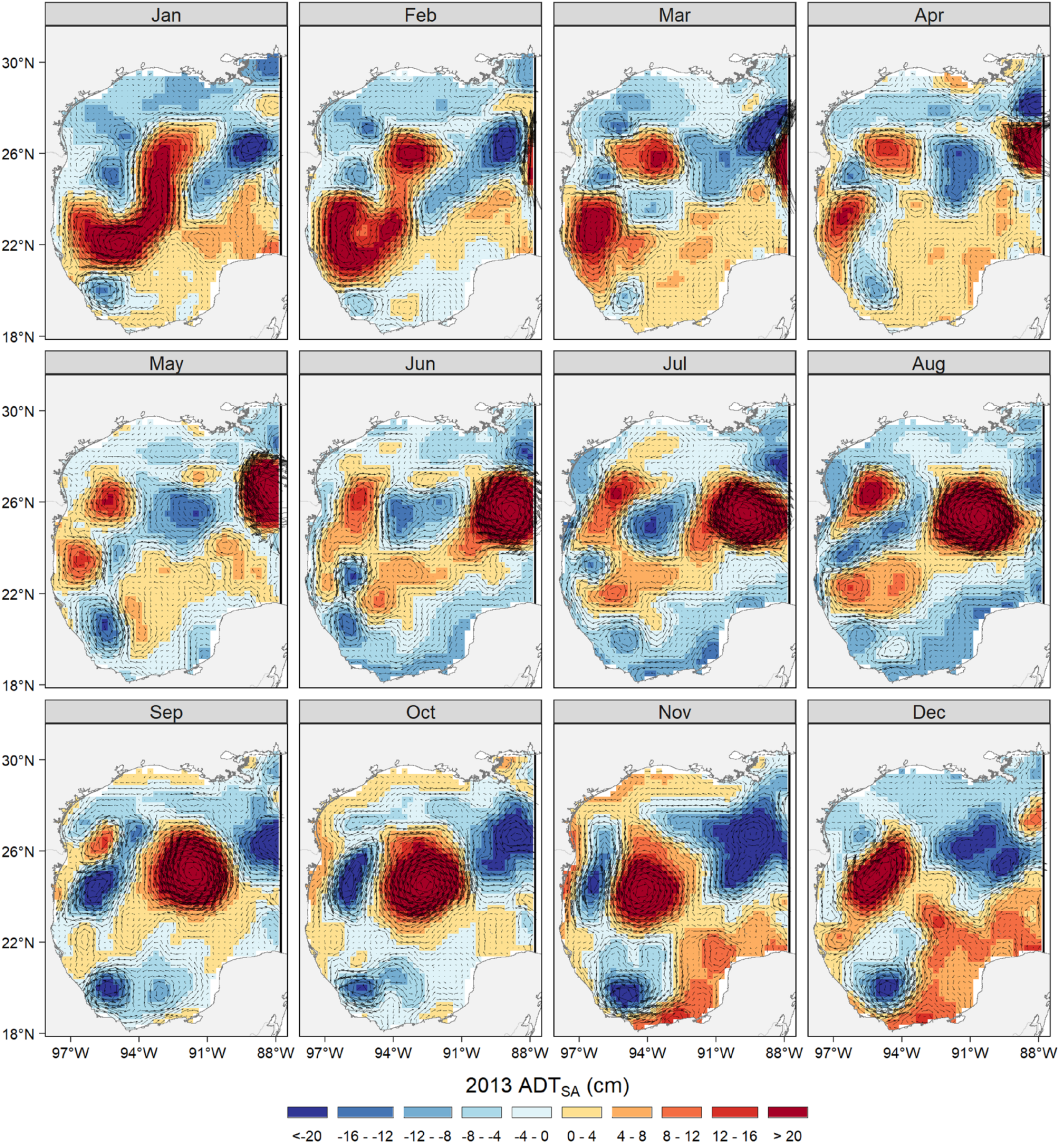
Map of absolute dynamic topography spatial anomalies (ADT_SA_) in 2013, as an example of an average year at the interannual scale. Black arrows represent the direction and magnitude of geostrophic velocities. Note how the variable captures cyclonic and anticyclonic eddies as they develop and move seasonally within the Gulf of Mexico. The maps were created with R’s package “ggplot2” (https://ggplot2.tidyverse.org/), using the coastlines from the Global Self-consistent, Hierarchical, High-resolution Geography Database (http://www.soest.hawaii.edu/pwessel/gshhg/).

The best model included the ADT_SA_ (index of mesoscale activity), bottom depth, and ADT_IA_ with third-degree polynomials as smoother functions, a monthly seasonal random effect, and the spatial effect. The second-best model had the same variables with second-degree polynomials as smoother functions and had a small difference in WAIC. The ADT_SA_ showed a clear seasonal variability and captured the development and movement of cyclonic and anticyclonic eddies within the GoM (Fig. 2). In spring–summer, large anticyclonic eddies (ADT_SA_ > 16 cm) separate from the Loop Current and travel southwestward, reaching the western GoM’s coast around early winter. In the southern GoM, the predominant negative ADT_SA_ suggested the presence of a persistent cyclonic circulation located west of 94°W. This cyclonic eddy is more intense in fall-winter (ADT_SA_ < -16 cm) than in spring–summer.

**Figure 3:**
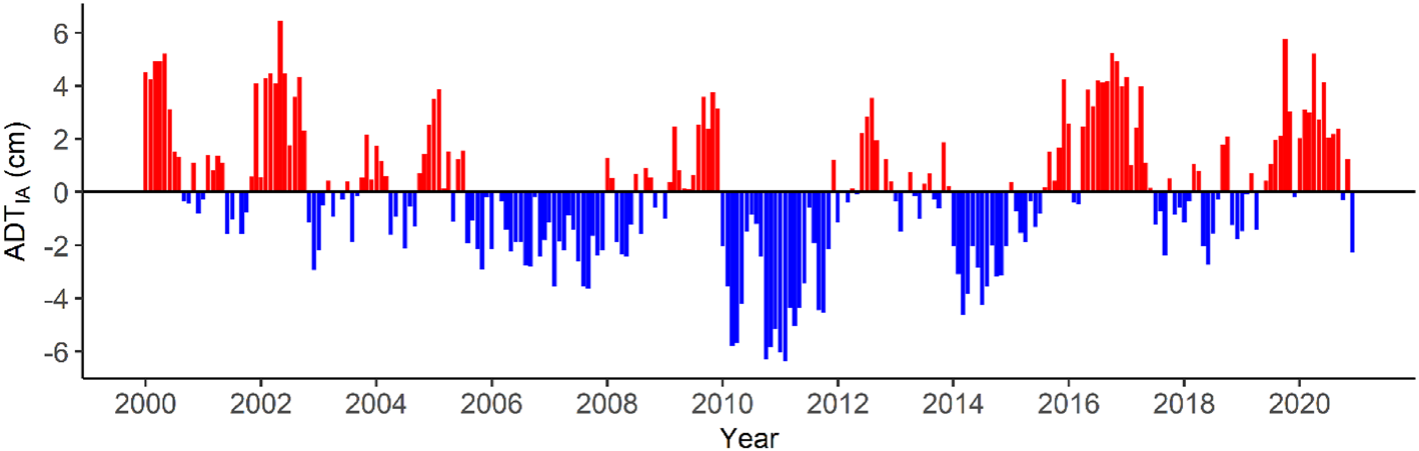
ADT interannual anomalies (ADT_IA_) in the GoM as residuals of the model portrayed in Supplementary Files: Fig. S3.

The time series of monthly means of ADT used to estimate the interannual anomalies showed a pronounced seasonal variability and a clear long-term increase in ADT spanning 2000–2020 (Supplementary Fig. S3). This trend represents an increase of 4.8 ± 0.24 cm per decade. The residuals (Fig. 3) showed an interannual variability with oscillation periods of around ~ 2 years. The lowest ADT_IA_ (< -5 cm) was observed in 2010–2011, while 2002 and 2019 exhibited the highest ADT_IA_ (~ 6 cm).

**Figure 4:**
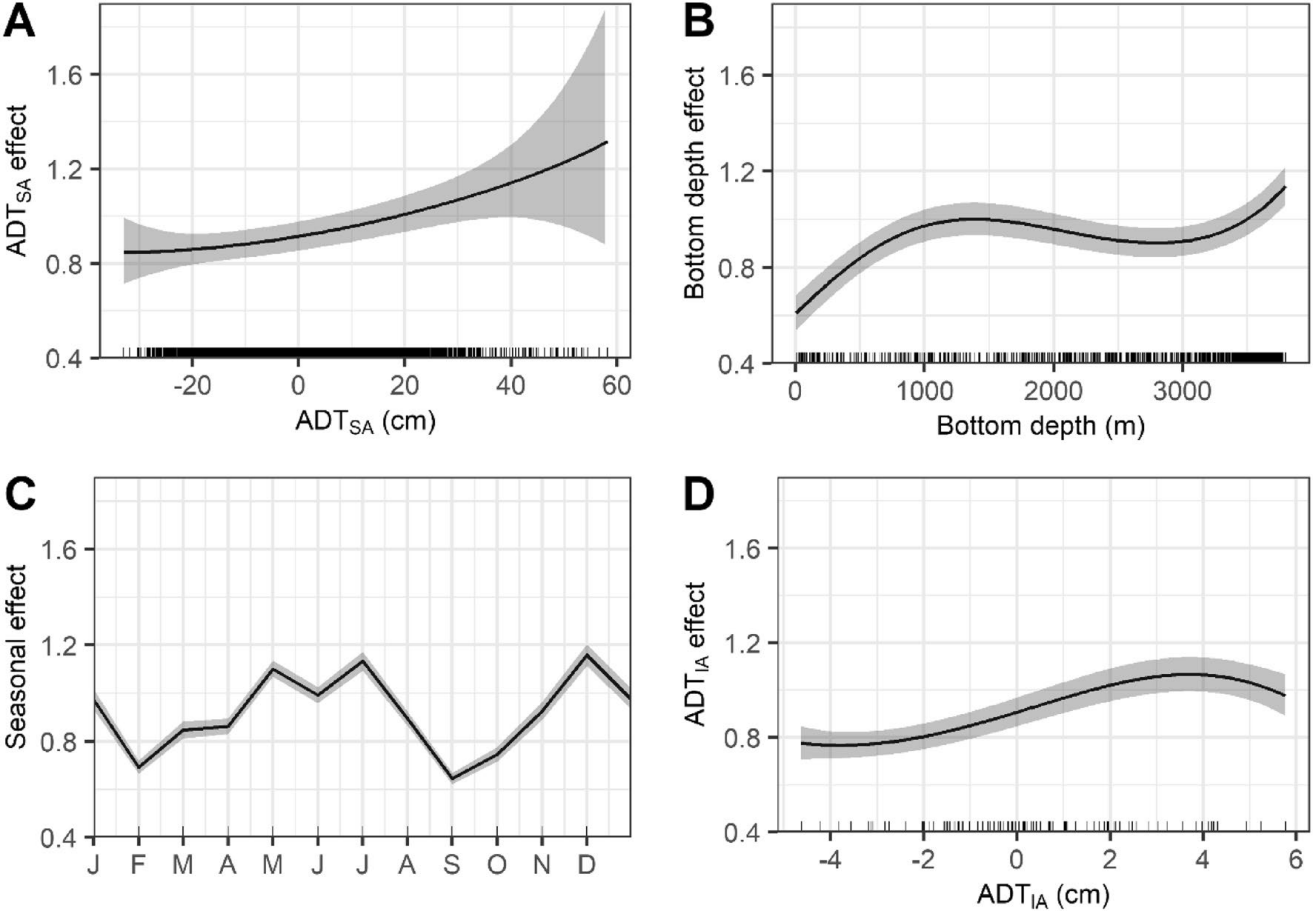
Partial effects of covariates on yellowfin tuna catch: A) absolute dynamic topography spatial anomalies (ADT_SA_), B) bottom depth, C) month (seasonal cyclic effect), D) absolute dynamic topography interannual anomalies (ADT_IA_). Gray-shaded areas represent 95%-credible intervals; the black line is the median effect. The rug lines on the X-axis are the values at each longline set.

The posterior partial effects of the best model’s covariates on yellowfin tuna habitat suitability (Fig. 4) showed a clear positive effect of ADT_SA_, with the highest predictions above + 32 cm, although with a higher degree of uncertainty (Fig. 4A) and a species’ distribution with a preference for oceanic waters with bottom depths > 1000 m (Fig. 4B). On the seasonal scale, the highest habitat suitability occurred in May–August (mid-summer) and December-January (early winter) (Fig. 4C). Interannual partial effects introduced as ADT_IA_ showed higher habitat suitability during years with positive values (up to 3.5 cm) but lower when they exceeded 4 cm (Fig. 4D). Therefore, we chose 2016 as representative of a positive year, 2013 as average, and 2014 as negative for portraying our model predictions (Fig. 3B; Fig. 6; Supplementary Figs. S12, S14, and S16).

**Figure 5:**
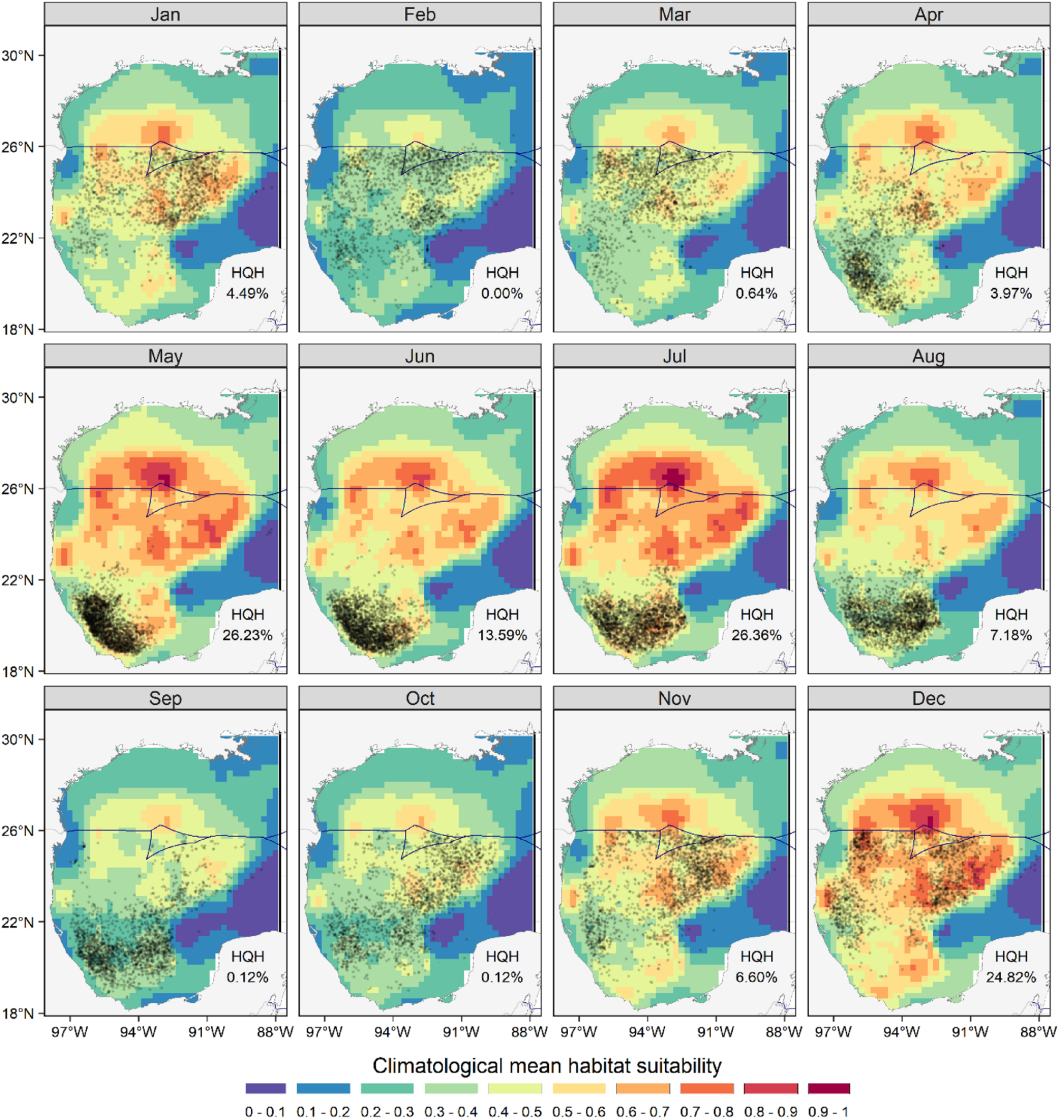
Climatological predictions of yellowfin tuna habitat suitability (colorimetric scale). The high quality (HQH) habitat percentage is the portion of the Gulf of Mexico with habitat suitability > 0.6. The blue line in the boundary between Mexican and U.S. Exclusive Economic Zones. Floating dots are the locations of all longline sets (black dots). The maps were created with R’s package “ggplot2” (https://ggplot2.tidyverse.org/), using the coastlines and political boundaries from the Global Self-consistent, Hierarchical, High-resolution Geography Database (http://www.soest.hawaii.edu/pwessel/gshhg/).

In general, climatological spatial predictions showed the highest values of habitat suitability (0.7–1) in oceanic waters of the central and northern GoM (Fig. 5). However, they also indicated that habitat suitability increases in the southern GoM during the summer (May–August) and fall (November–December), as well as a gradual shift towards the northern GoM in the following months (January–April). Interestingly, high suitability values (> 0.6) in the southern GoM, within the Bay of Campeche (south of 22° N), were associated with negative ADT_SA_ (− 20 to − 3 cm; Supplementary Files: Fig. S19), close to the continental shelf off Veracruz and Tabasco. In contrast, during the rest of the year, high habitat suitability values and catches occurred in waters with mostly positive ADT_SA_, between − 20 and 40 cm. High suitability values (> 0.7) were also predicted during the summer months in the northern GoM. However, the standard deviations of the predictions showed high uncertainty in that area, given the lack of observations for the model (Fig. 5).

**Figure 6:**
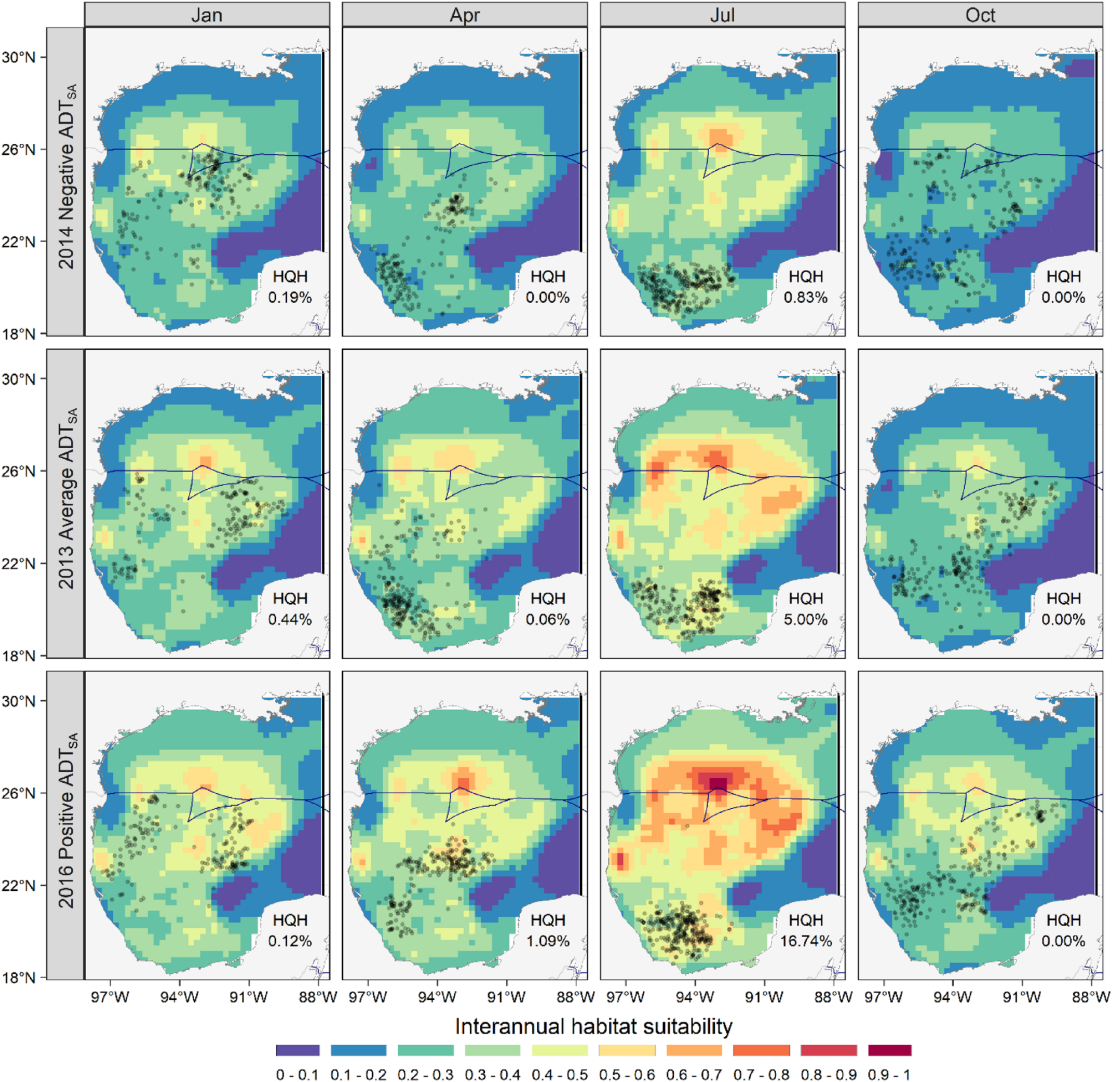
Predictions of yellowfin tuna habitat suitability for years (strip labels) with negative, average, and positive interannual anomalies of the mean absolute dynamic topography (i.e., ADT_IA_). The high-quality habitat (HQH) percentage represents the portion of the Gulf of Mexico with habitat suitability > 0.6. The blue line is the boundary between Mexican and U.S. Exclusive Economic Zones. Floating dots are the locations of longline sets. The maps were created with R’s package “ggplot2” (https://ggplot2.tidyverse.org/), using the coastlines and political boundaries from the Global Self-consistent, Hierarchical, High-resolution Geography Database (http://www.soest.hawaii.edu/pwessel/gshhg/).

The spatial extent of high-quality habitat during 2016 (positive ADT_IA_) was broader (up to 16%), showing more localized predictions than those of 2013 (average ADT_IA_) or 2014 (negative ADT_IA_). An extensive reduction in suitable areas was predicted under average and negative ADT_IA_, with high-quality habitat ranging between 0 and 5%. However, high-quality habitat predictions occurred mainly in the central and northern GoM (Fig. 6).

## Discussion

Our results suggest that the spatiotemporal distribution of yellowfin tuna is linked mainly to mesoscale oceanographic features in oceanic waters of the GoM, especially towards boundary regions between cyclonic and anticyclonic eddies, but with a preference for the latter. The species’ habitat suitability responded positively to intermediate and positive values of ADT_SA_. The latter indicates a deeper thermocline and often anticyclonic circulation (convergent zones), whereas negative ADT_SA_ values mean thermocline shoaling and cyclonic circulation (divergent zones)^37,38^. In contrast to other dynamic variables such as SST or CHL, ADT reflects changes in the structure of the entire water column^39^ since it responds to the overall density and, therefore, the volume, triggered by pycnocline deepening or shoaling, as well as by stratification or mixing^38,40^. Regions with deep pycnocline exhibit warm and less dense water in the upper layer, which increases the volume of the water column, and thus, the ADT. In contrast, the diverging regions show deep, cold, and denser waters reaching the upper layer, shoaling the pycnocline, and decreasing the volume and height of the water column^37,41^.

Higher habitat suitability predictions in oceanic waters occurred mostly towards positive ADT_SA_, which implies a preference for areas with a deeper thermocline, specifically those associated with Loop-Current-derived anticyclonic eddies, also known as warm-core eddies. The convergence effect of anticyclonic eddies transports warm and oxygen-rich water from the surface into the mesopelagic zone and drives zooplankton and micronekton from the eddy’s periphery towards its core, aggregating biomass^42,43^. There, the consequent increase in mesopelagic prey abundance may attract top predators such as tunas and sharks, leading to the formation of ecological hotspots where these predators aggregate and prey on small organisms^42,44^. In addition, warm and oxygen-enriched waters along the cores of anticyclonic eddies may alleviate physiological constraints for adult yellowfin tuna during deep-feeding dives, like what has been observed in white sharks (*Carcharodon carcharias*)^45^.

On the other hand, at the edges of anticyclonic eddies, vertical shear leads to upward vertical nutrient transport, supporting higher productivity and biomass, as well as zooplankton aggregation^46,47^. Previous studies have pointed to a finer-scale spatial utilization of these mesoscale features by yellowfin tuna. Specifically, the species that use the margins of anticyclonic eddies during their seasonal migrations within the GoM likely allows them to feed in areas of high prey aggregation^7^. These edges could also present an energetic advantage since their geostrophic velocities are higher (Fig. 2), which would reduce drag for large predators during locomotion^48^, helping them to exceed the energetic demand associated with swimming during foraging or migration.

During most of the year, the catches occurred mainly in the central GoM and were associated with positive ADT_SA_ indicative of anticyclonic eddies (Supplementary Fig. S19). However, from May to August, catches were associated with areas of negative ADT_SA_ (− 20 to − 3 cm; Supplementary Fig. S19), which suggests a preference of the species for cooler, higher-nutrient waters typical of cyclonic eddy cores^49,50^ during the strongly stratified summer. In addition, freshwater inflow from the Grijalva-Usumacinta River System, which is highest in June-December, increases the concentration of nutrients in the oceanic region, enhancing regional productivity^28,51^. All these processes produced a seasonal variation in yellowfin tuna habitat suitability and considering that this fishery is mainly based on adult individuals (Supplementary Fig. S4), it could be related to the species' reproductive behavior. In Mexican waters, the presence of males and females at all stages of gonadal maturation occurs throughout the year^52^. However, the summer peak in catch per unit effort (Supplementary Fig. S5) off the coast of Veracruz is likely a consequence of the aggregation of the species in the area for spawning and foraging, as suggested by the occurrence of females mainly in advanced maturity, pre-spawning, and spawning stages in May-August^52^. Hence, the seasonal preference for specific regions of the GoM may favor larval survival and recruitment.

Our models predicted high habitat suitability for yellowfin tuna in the northern GoM throughout the year, although catch data were limited to the Mexican Exclusive Economic Zone (Fig. 5). This region has been increasingly recognized as important habitat, not only for foraging individuals captured in the southern GoM^20^, but also as a spawning ground for yellowfin tuna^6^, as well as for other tunas^32^, swordfish (*Xiphias gladius*)^53^, and billfishes^54^. Tagging studies also suggest that yellowfin tuna exhibits some degree of residency in the northern GoM^17^, although there is also evidence of connectivity with the southern GoM^21^. The northern GoM is highly productive and dominated by the Loop Current and the formation of anticyclonic eddies mainly in summer (July–September), due at least in part to the forcing of seasonal winds in the Caribbean Sea and GoM^55,56^. These mesoscale eddies interact with the inflow from the Mississippi-Atchafalaya River System^57^ leading to offshore transport of nutrient-enriched waters and relatively high primary and secondary production^28^. In addition, higher wind-driven mixing during the winter months leads to higher surface and integrated chlorophyll-*a* concentrations in the northern GoM, compared to those of the central and southern GoM^58,59^. Intrinsic isotope tracers have also shown that both the northern and southern GoM are important foraging regions for yellowfin tunas caught by the Mexican longline fleet^20^. By the end of the summer, the Mexican fleet moves northward, close to U.S. waters, presumably “following” the abundance of yellowfin tuna^12,20^, which agrees with our climatological predictions of high habitat suitability for both regions.

Our best model also included the interannual variability of ADT as an important predictor. This scale of variation in the GoM is spatially and temporally dynamic^24,58^, and is likely related to the level of intrusion of the Loop Current into the northern GoM each year, and the frequency with which eddies are released^24^. During the highest ADT_IA_ (i.e., 2016), the GoM exhibited a greater extent of high habitat suitability for yellowfin tuna compared to those with average (i.e., 2013) or lowest (i.e., 2014) interannual anomalies (Fig. 6). Since 2003, the Loop Current has shown a greater level of intrusion, which implies that larger volumes of oligotrophic waters from the Caribbean Sea have been transported to the central and western GoM^56^. This would also increase the number of anticyclonic eddies formed per year^24^ and, consequently, the positive long-term increase in the mean annual ADT within the GoM we observed (Fig. 3). Therefore, the spatial dynamics of the Loop Current and its eddies could be an important determinant of the interannual variations in the habitat suitability of yellowfin tuna, as it affects oceanographic conditions throughout the GoM^24,56^. Nevertheless, it should be noted that positive ADT_IA_, as well as the positive long-term trend of ADT, may be attributed to more anticyclonic activity in the GoM^56^ and an increase in the thermocline depth or surface warming and vertical stratification over time^58^, which could result in lower primary production. Interestingly, the positive partial effects of high ADT_IA_ on yellowfin tuna catches occur only up to + 3.5 cm. Above that threshold, the effect was negative (Fig. 4D). Even though the ADT was not included in the best model, its positive trend may lead to future shifts in suitable habitats for yellowfin tuna in the GoM and calls for further research effort.

The spatial random effect map (Supplementary Fig. S18) showed a similar pattern to the habitat suitability predictions, which suggests that the variability of the fishing data for yellowfin tuna could not be explained only by the environmental predictors in the model^60^. Including the spatial random effect improves the fits of the models and may reflect the effect of other unconsidered factors that are affecting the species' spatial distribution.

Although high habitat suitability predictions occurred mainly in waters deeper than 1000 m, there were some exceptions. In addition to the Bay of Campeche in the southern GoM, high values were also predicted off the midwestern continental shelf (Fig. 5), influenced by the periodic arrival of Loop-Current-derived anticyclonic eddies^26^ and by upwelling-favorable winds over the shelf and slope from April to August^61^. Average to high values of habitat suitability were also predicted close to the northern coast of the GoM, influenced by the Mississippi-Atchafalaya River System, particularly during early summer (May–June) and winter (November–December) (Fig. 5). This region has been described as important for both larvae and adult yellowfin tuna^17,21,62^. The average habitat suitability values predicted for this area may be related to the offshore transport of low-salinity and nutrient-rich waters from the Mississippi River^57^. This transport enhances primary production and biomass aggregation in cyclonic and anticyclonic mesoscale eddies, respectively^28,57^.

Our model results identified areas of high habitat suitability for yellowfin tuna and provide a baseline for evaluating future impacts of anthropogenic and natural disturbances common to this region (e.g., oil spills, hurricanes) and for predicting the potential effects of climate change on the species. Understanding the effects of environmental conditions and identifying the essential habitats of highly migratory fishes is critical in ecosystem-based fisheries management, especially when their distributions are highly dynamic^63^. This study’s analytic and ecological framework might help develop dynamic fisheries management strategies between Mexico and the U.S.

## Conclusions

This study improved the understanding of the spatiotemporal distribution of yellowfin tuna in the GoM using fishery-dependent data. Further studies might consider encompassing fishery data from all commercial longline fisheries that target yellowfin tuna in the GoM.

The Bayesian INLA framework allowed us to incorporate several sources of variability in the competing models, including spatial and temporal effects. By providing realistic estimations of specific associations between yellowfin tuna catches and oceanographic conditions, we gained a deep understanding of the main drivers of yellowfin tuna distribution. As it was shown, high habitat suitability for yellowfin tuna is strongly linked to the mesoscale structure and dynamics of the water column they inhabit.

In addition, our model predictions highlight the importance of the northern GoM as an essential year-round habitat for yellowfin tuna. However, monthly predictions showed that the habitat suitability shifts to southern GoM during the summer, which may be associated with the aggregation of yellowfin tuna in the area for spawning. The Bayesian INLA framework can be used more widely to complement alternative models based on fishery-independent surveys, which are less biased but limited spatially and temporally.

## Methods

All data processing, analyses, and graphics described below were performed in R, version 4.1.2^64^.

### Fishery data

Yellowfin tuna catch and effort data were collected by fishery observers onboard Mexican commercial longline vessels that operate year-round within Mexico’s Exclusive Economic Zone in the GoM, limited eastward to 88° W (Fig. 1). Fishery observers registered 100% of fishing pelagic longline operations as part of the National Program for Tuna Exploitation and Dolphin Protection (https://www.fidemar.org). The database used in this study included the number of yellowfin tuna caught, date, geographic position, size, sex, and fishing effort (i.e., number of hooks) for the longline sets deployed from 2012 to 2019 (n = 26,515 sets). However, sex information was not available for all the sets. We discarded sets with wrong or missing geographic coordinates (n = 3). In addition, an exploratory analysis of the temporal variability in the catch per unit effort (CPUE, fish per 100 hooks), size and sex distribution of yellowfin tuna was performed.

### Environmental data

We considered the following dynamic environmental variables as potential predictors of habitat suitability for yellowfin tuna: sea surface temperature (SST, °C), sea surface chlorophyll-*a* concentration (CHL, mg m^−3^), and the absolute dynamic topography of the ocean surface (ADT, cm) (Table 2). These variables were selected as they have been previously associated with the abundance and distribution of several marine species^65–67^ and because of their adequate spatial and temporal coverage to pair with our fisheries data. In addition, since yellowfin tuna prefers the pelagic oceanic environment^65^, we included the bottom depth (m) deterministically in all model structures.

**Table 2:** Environmental data used to model the spatio-temporal habitat suitability of yellowfin tuna.

Variable	Units	Original spatial and temporal resolution	Source
Sea surface temperature	°C	0.041°/daily	National Oceanic and Atmospheric Administration (NOAA)’s Advanced Very High-Resolution Radiometer (AVHRR) Pathfinder 5.3 database (https://www.ncei.noaa.gov/products/avhrr-pathfinder-sst)^69,70^
Absolute dynamic topography	m	0.25°/daily	Copernicus Marine Environment Monitoring Service (https://marine.copernicus.eu/)
Sea surface chlorophyll-*a* concentration	mg m^−3^	0.041°/8-d composite	Derived from MODIS-Aqua sensor. Ocean Color Portal (https://oceancolor.gsfc.nasa.gov/)
Depth	m	0.008°	Satellite Geodesy dataset V11.1 (https://topex.ucsd.edu/pub/global_topo_1min/)^71^

Original data values of dynamic environmental variables include several scales of temporal variability that typically mask processes such as the mesoscale (e.g., eddies and thermal fronts). To identify these spatial variations, we estimated spatial anomalies of each variable (i.e., SST_SA_, ADT_SA_, and CHL_SA_). First, for any given moment in the dataset (i.e., a time layer), we estimated the mean value of the original variable within the study area polygon (Fig. 1). Then, we subtracted that mean from the original value at each cell (i.e., pixel) within the polygon during that exact moment. This procedure effectively removes all scales of time variations because the resulting mean values change at the original temporal resolution of the variable, keeping only the spatial gradients (e.g., Supplementary Fig. S1). These environmental spatial anomalies have proven useful in addressing habitat suitability predictions for dolphins in the northern Humboldt Current System^68^.

All variables, including the spatial anomalies, were scaled to a 0.25° resolution before pairing each longline set to its closest variable cell in space and time. Averaging was used to reduce the original spatial resolution of all variables to 0.25-degree cells from the original resolution (Table 2). The assigned values represented 10-day averages of SST and ADT, and 16-day averages of CHL, before the logline set. CHL values were transformed to a logarithmic scale.

Finally, to examine the potential effects of interannual variability on yellowfin tuna habitat suitability, we estimated the residuals of a time series analysis of monthly means of SST (2000–2021) and ADT (2000–2020) within the study area, which included the seasonal variability and the long-term trend. We preferred this approach instead of using common indices of global climate variability, this allows the determination of the dynamic structure and changes in the mesoscale activity in the region. These residuals would represent the interannual anomalies, hereafter SST_IA_ and ADT_IA_, and were included as additional potential covariates in the competing model structures (Supplementary Figs. S2 and S3).

### Modeling approach

Before running the models, we looked for correlations among predictors and proposed possible likelihoods for the response variable. The presence of outliers was inspected (Supplementary Files: Figs. S7 and S8)^60^. The former was evaluated with Pearson’s correlation coefficients using the R package *corrplot*, and collinearity was computed using the generalized variance inflation factor (GVIF). When a pair of variables had high correlation values (Pearson correlation |r|> 0.6) or high variance inflation factor (GVIF > 3), only one of them was included in the same competing model (Supplementary Fig. S9 and Table S2). Scatterplots of yellowfin tuna count against each potential predictor were used to explore nonlinear relationships. Given the complexity of some relationships between continuous covariates and the response variable, we used splines and polynomial functions as smoothing techniques within some competing models.

The number of yellowfin tuna caught on a longline set was considered a count variable; hence, it was used as a response variable. This allowed us to test different likelihoods, like Poisson and negative binomial with logarithmic link functions^72^. Since the number of yellowfin tuna caught in each longline set depends on the effort applied (i.e., the number of hooks), we included an *offset* variable (*E*) that balanced the response variable by introducing a known component of the likelihood’s mean:

1 $${E}_{i}=\frac{\sum {YFT}_{i}}{\sum {H}_{i}}\cdot {H}_{i}\hspace{1em}i=1,...,n$$

where *YFT* is the number of fish caught, and *H* represents the number of hooks deployed in each longline set, *i*. A Bayesian hierarchical modeling approach^73^ was used to obtain our results in terms of probabilities. Since we needed to test several competing structures and random effects, posterior distributions of model parameters were estimated using the integrated nested Laplace approximation (INLA)^33^ and the package “*INLA*” in R^74^. This approximation is less computationally demanding than Markov chain Monte Carlo methods and allows for rapid inference and high regression complexity, such as cyclic temporal and triangular spatial autocorrelation effects.

To account for spatial dependency between observations, we included a spatial autocorrelation random effect (*W*) in all competing models. This was done by applying stochastic partial differential equations (SPDE)^33^ to approximate the Gaussian Markov random field with the Matérn covariance function^36,60^. This involves dividing the study area into an irregular spatial triangular mesh (i.e., the *Delaunay triangularization*) covering the entire geographic coordinates of the longline sets (Supplementary Fig. S10)^60^. After accounting for the alternative combinations of environmental variables, smoothing terms, and likelihoods, 228 competing models were tested. Equation 2 portrays the general structure of the models using an example with a negative binomial likelihood (intercept and residuals are omitted):

2 $$ \begin{gathered} \mu_{i} = \left[ {\mathop \sum \limits_{{\begin{array}{*{20}c} {j = 1} \\ {k = 1} \\ \end{array} }}^{{\begin{array}{*{20}c} {j = ns} \\ {k = nc} \\ \end{array} }} \beta_{j} \cdot X_{k,i} } \right] + W_{i} + \gamma \cdot M_{i} \quad i = 1,...,n \hfill \\ W_{i} \sim N\left( {0,\sum } \right) \hfill \\ \end{gathered} $$

where the expected number of yellowfin tuna ($\mu$) in each longline set (*i*) is a function of several combinations (*k*) of environmental variables (*X*). *β* represents any smoothing function or polynomial applied to each covariate. The term ${W}_{i}$ in the model represents the spatially structured random effect for each longline set *i*, whose hyper distribution is Normal with mean 0 and a covariance matrix ∑ that accounts for the spatial autocorrelation among the observations at nearby locations^60^. *γ* represents a seasonal (i.e., cyclical) random effect of the month (*M*). Default Gamma distributions were used as vague priors for all unknown parameters. Model selection was based on the lowest Watanabe-Akaike Information Criterion (WAIC).

### Habitat suitability predictions

Since our focus was understanding and quantifying the species-environment relationships, habitat suitability maps were produced from the best model's posterior medians and standard deviations of yellowfin tuna catch predictions, scaled to 1. Monthly predictions were made for specific years and for the climatology (2012–2019) of the covariates. A percentage of high-quality habitat (habitat suitability ≥ 0.6) was estimated relative to the total study area for each prediction map. Finally, we mapped predictions for years with the highest, lowest, and average ADT_IA_ for understanding interannual responses of habitat suitability.

### Supplementary Information

Supplementary Information.

## Data Availability

Yellowfin tuna catch data may be available upon request to Armando Diaz (adiaz@cicese.mx) and the review of the research proposal. Environmental data and non-systematic yellowfin tuna records are freely available from the links provided within the manuscript.
