# Supplementary material for: Mesoscale activity drives the habitat suitability of yellowfin tuna in the Gulf of Mexico
Source: Sci Rep. 2024 Apr 8;14:8256. doi: 10.1038/s41598-024-58613-7 (PMC11001853; doi:10.1038/s41598-024-58613-7)
Supplement: Supplementary file 1 — Supplementary Information. [file 41598_2024_58613_MOESM1_ESM.pdf]

Mesoscale activity drives the habitat suitability of yellowfin tuna in the Gulf of Mexico

Zurisaday Ramírez-Mendoza[1], Oscar Sosa-Nishizaki[1], Mario A. Pardo[2,*], Sharon Z. Herzka[3], R. J. David Wells[4,5], Jay R. Rooker[4,5], Brett J. Falterman[6], Michel J. Dreyfus-León[7]

SUPPLEMENTARY INFORMATION

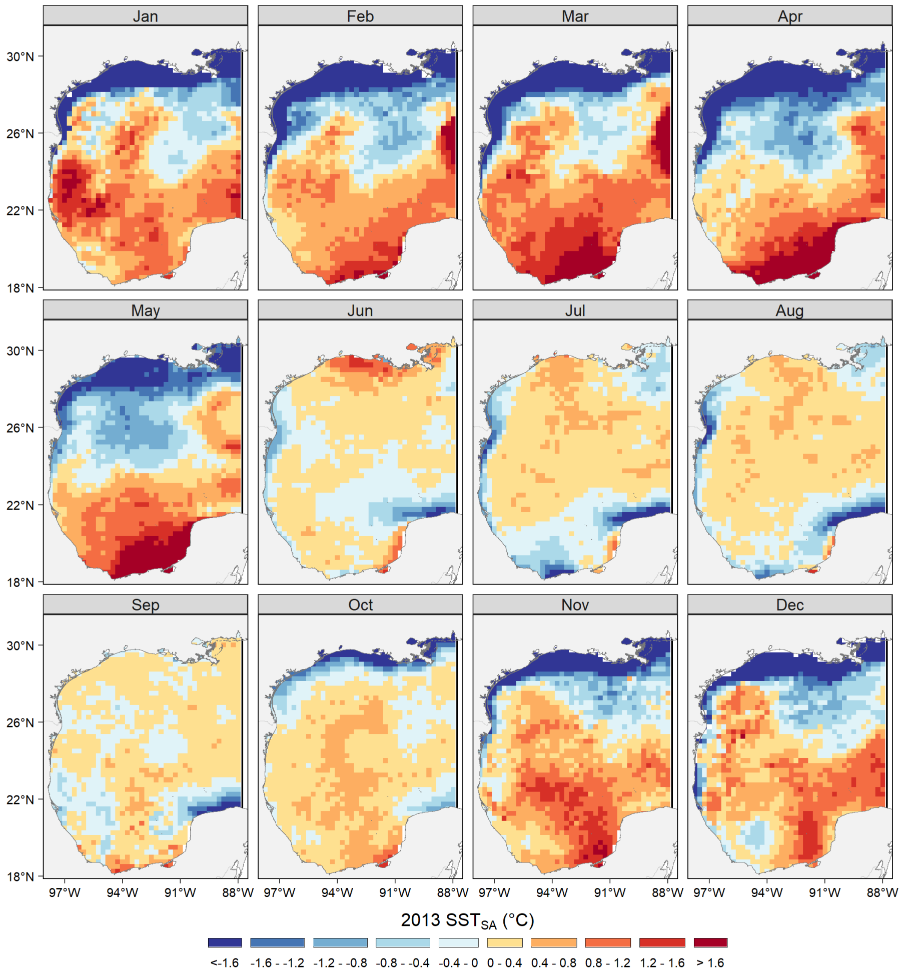

Fig. S1. Map of sea surface temperature spatial anomalies (SST$_{SA}$) in 2013 as an example of an average year at the interannual scale. The maps were created with R's package "ggplot2" (https://ggplot2.tidyverse.org/), using the coastlines from the Global Self-consistent, Hierarchical, High-resolution Geography Database (http://www.soest.hawaii.edu/pwessel/gshhg/).

To examine the effects of interannual variability on the yellowfin tuna habitat suitability, we calculated the monthly means and standard deviation of sea surface temperature (2000-2021) and absolute dynamic topography of the ocean surface (2000-2020). Then, these monthly means were used to estimate the interannual anomalies, hereafter SST_{IA} and ADT_{IA}, using the following regression model:

$$\mu_i = \alpha + \beta * Ti + \gamma(M_i)$$

where μ_i is the monthly sea surface temperature or absolute dynamic topography along the time series (i= 1…n), T is the month count (n=264 for the SST, and n=252 for ADT), β is the coefficient representing the long-term linear time trend, and γ represents a seasonal (i.e., cyclical) random effect of the month (M). The interannual anomalies of each variable (SST_{IA} and ADT_{IA}) were obtained by subtracting the model's predictions from the observed data for a given location and were assigned to each longline set as additional potential predictors of the yellowfin habitat suitability.

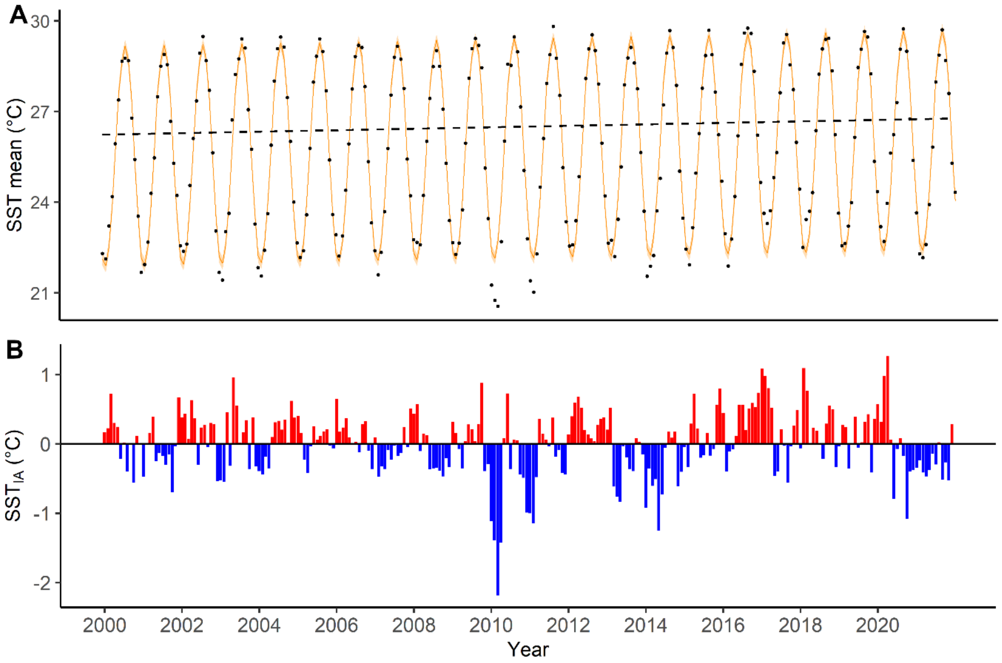

Fig. S2. A) Time series model of monthly means of the sea surface temperature (SST; black dots) in the Gulf of Mexico (GoM). The median model prediction (orange line) and the 95%-credible intervals (orange shaded area) include both the seasonal random effects and the long-term linear trend (black dashed line). B) SST interannual anomalies (SST_{IA}) in the GoM, which represent the residuals of the model portrayed in panel A.

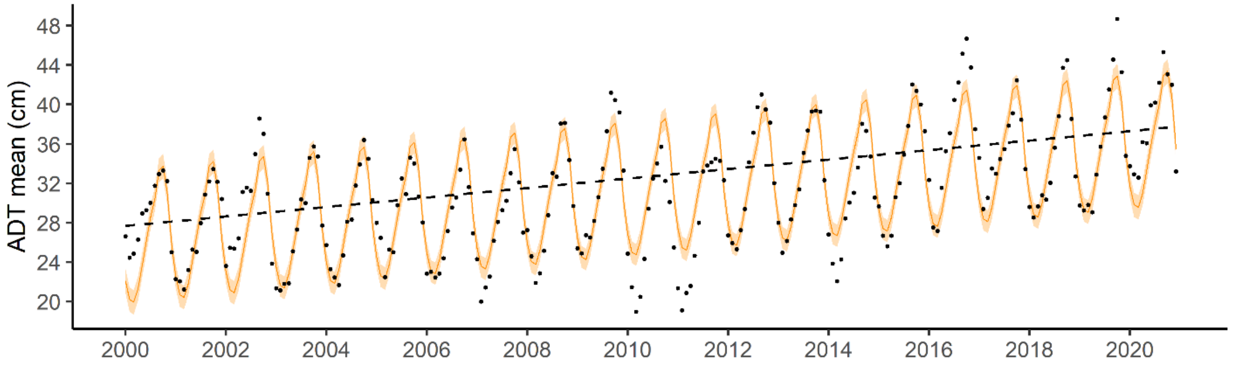

Fig. S3. Time series model of monthly means of absolute dynamic topography (ADT; black dots) in the Gulf of Mexico (GoM). The median model prediction (orange line) and the 95%-credible intervals (orange shaded area) include both the seasonal random effects and the long-term linear trend (black dashed line).

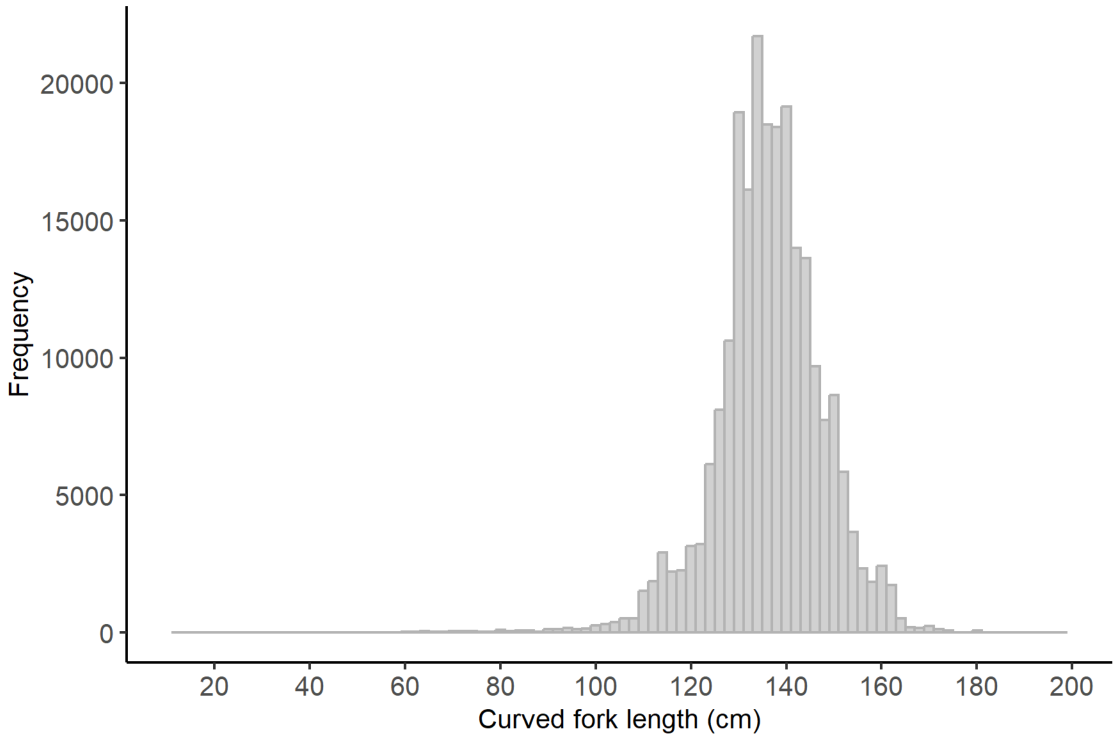

Fig. S4. Frequency distribution of the curved fork length (cm) of yellowfin tuna caught by the Mexican fishery.

Table S1. Annual sex ratio of yellowfin tuna caught by the Mexican longline fleet in the Gulf of Mexico between 2012 and 2019.

Year	Number of individuals	Sex ratio M: F
**2012**	**34,178**	**1.27**
**2013**	**26,876**	**1.27**
**2014**	**26,714**	**1.18**
**2015**	**25,381**	**1.21**
**2016**	**36,193**	**1.22**
**2017**	**34,310**	**1.22**
**2018**	**24,288**	**1.19**
**2019**	**20,094**	**1.54**

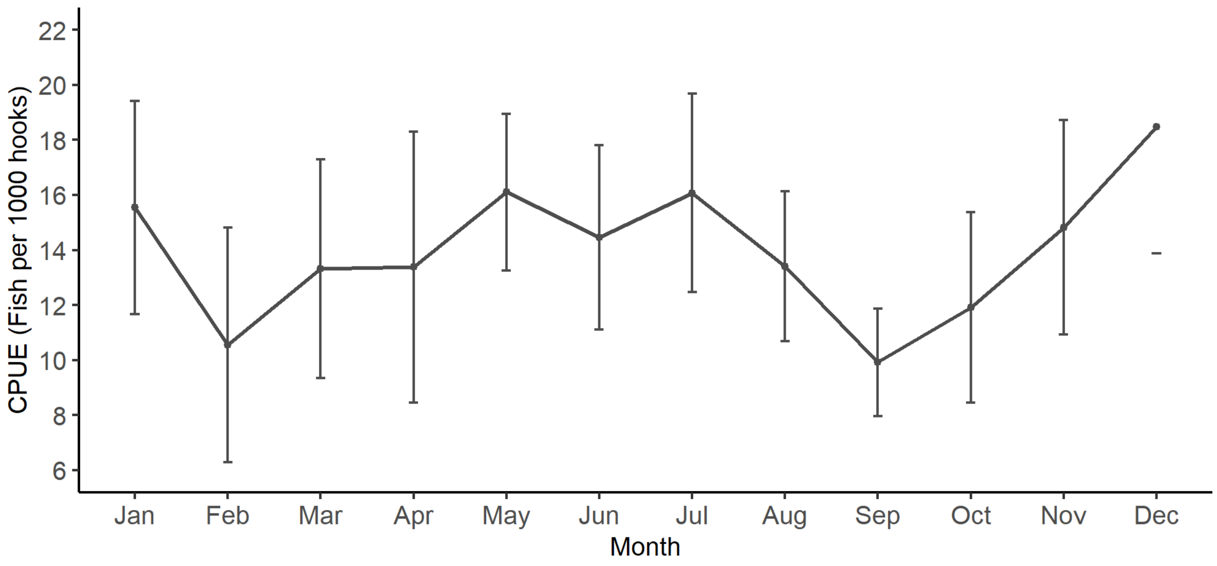

Fig. S5. Monthly average (±SD) of the catch per unit effort (fish per 1000 hooks) of yellowfin tuna caught by the Mexican longline fleet in the Gulf of Mexico.

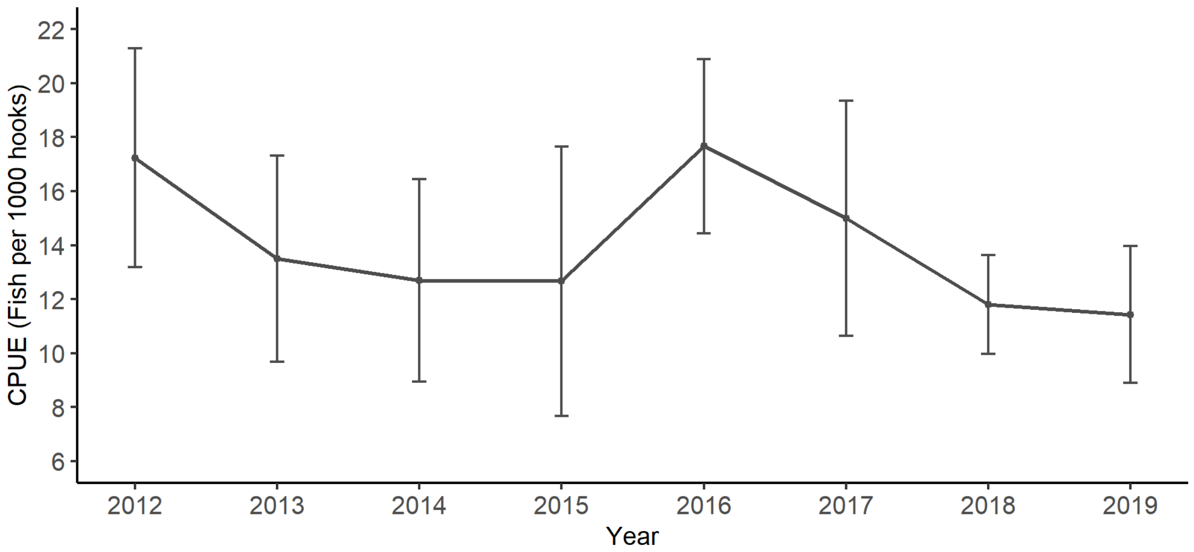

Fig. S6. Annual average (±SD) of the catch per unit effort (fish per 1000 hooks) of yellowfin tuna caught by the Mexican longline fleet in the Gulf of Mexico.

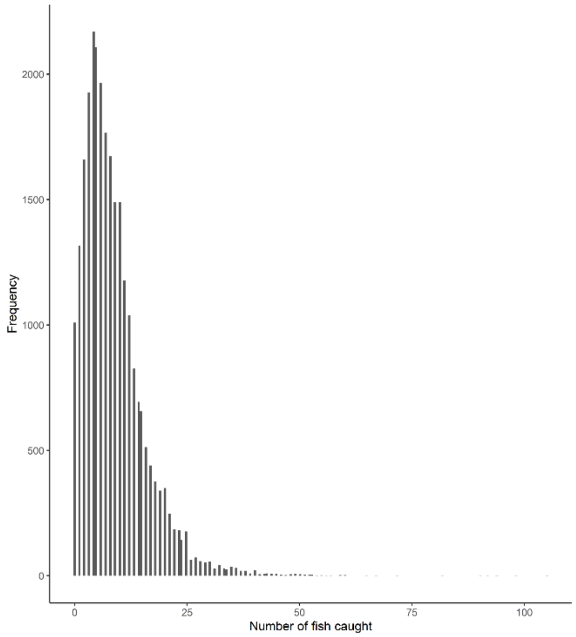

Fig. S7. Frequency distribution of the number of yellowfin tuna caught in each longline set.

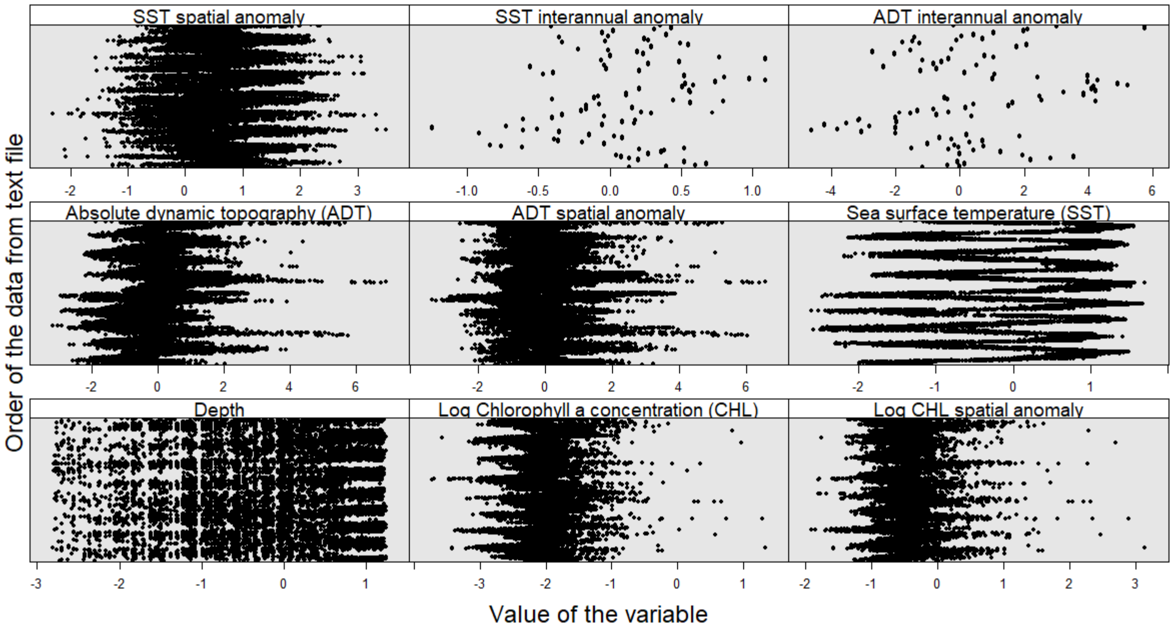

Fig. S8. Cleveland dotplot used to visualize outliers. In this graph the row number of an observation is plotted vs. the observation value.

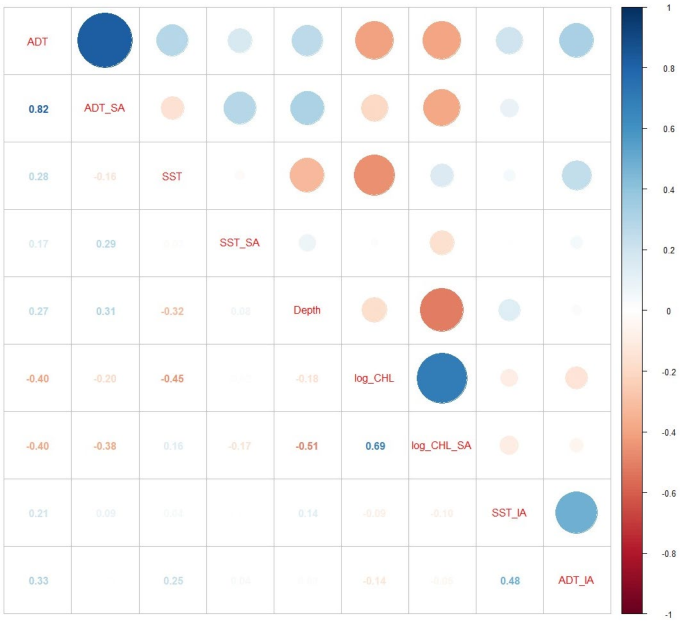

Fig. S9. Pearson's correlation coefficient values between environmental variables. The magnitude of the correlation is represented in the upper right by red circles (negative correlation) and blue circles (positive correlation).

Table S2. Analysis of collinearity between variables derived by calculating the Generalized Variance Inflation Factor (GVIF). Predictor acronyms are: SST= sea surface temperature, SST_{SA}= Sea surface temperature spatial anomalies, ADT= absolute dynamic topography, ADT_{SA} = absolute dynamic topography spatial anomalies, CHL= sea surface chlorophyll-*a* concentration, CHL_{SA}= sea surface chlorophyll-*a* concentration spatial anomalies, ADT_{IA} = absolute dynamic topography interannual anomalies, SST_{IA}= sea surface temperature interannual anomalies.

Variable	GVIF
SST	7.4626
SST_{SA}	1.3291
ADT	10.3507
ADT_{SA}	8.8472
CHL	6.8451
CHL_{SA}	6.9057
Bottom depth	1.5862
ADT_{IA}	2.1395
SST_{IA}	1.3512

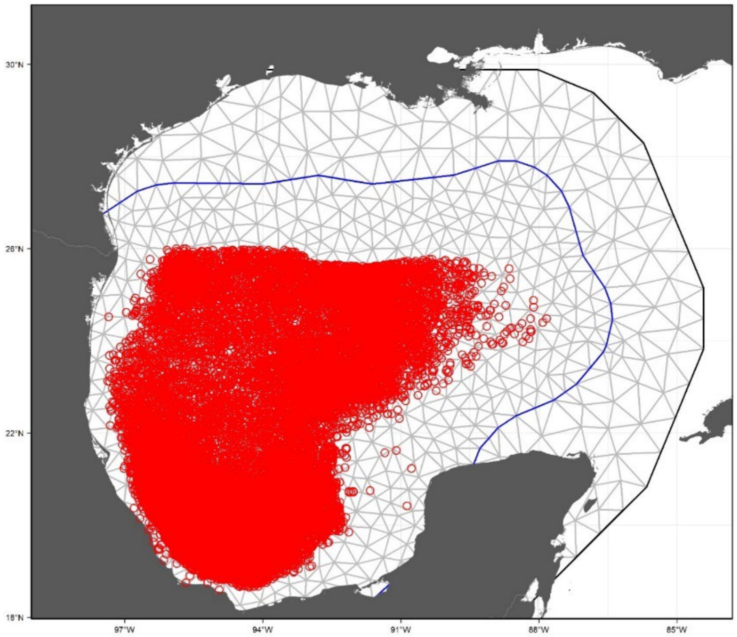

Fig. S10. Delaunay triangulation. Red points represent the fishing locations of the Mexican longline fishery (2012-2019). The map was created with R's package "ggplot2" (https://ggplot2.tidyverse.org/), using the coastlines from the Global Self-consistent, Hierarchical, High-resolution Geography Database (http://www.soest.hawaii.edu/pwessel/gshhg/).

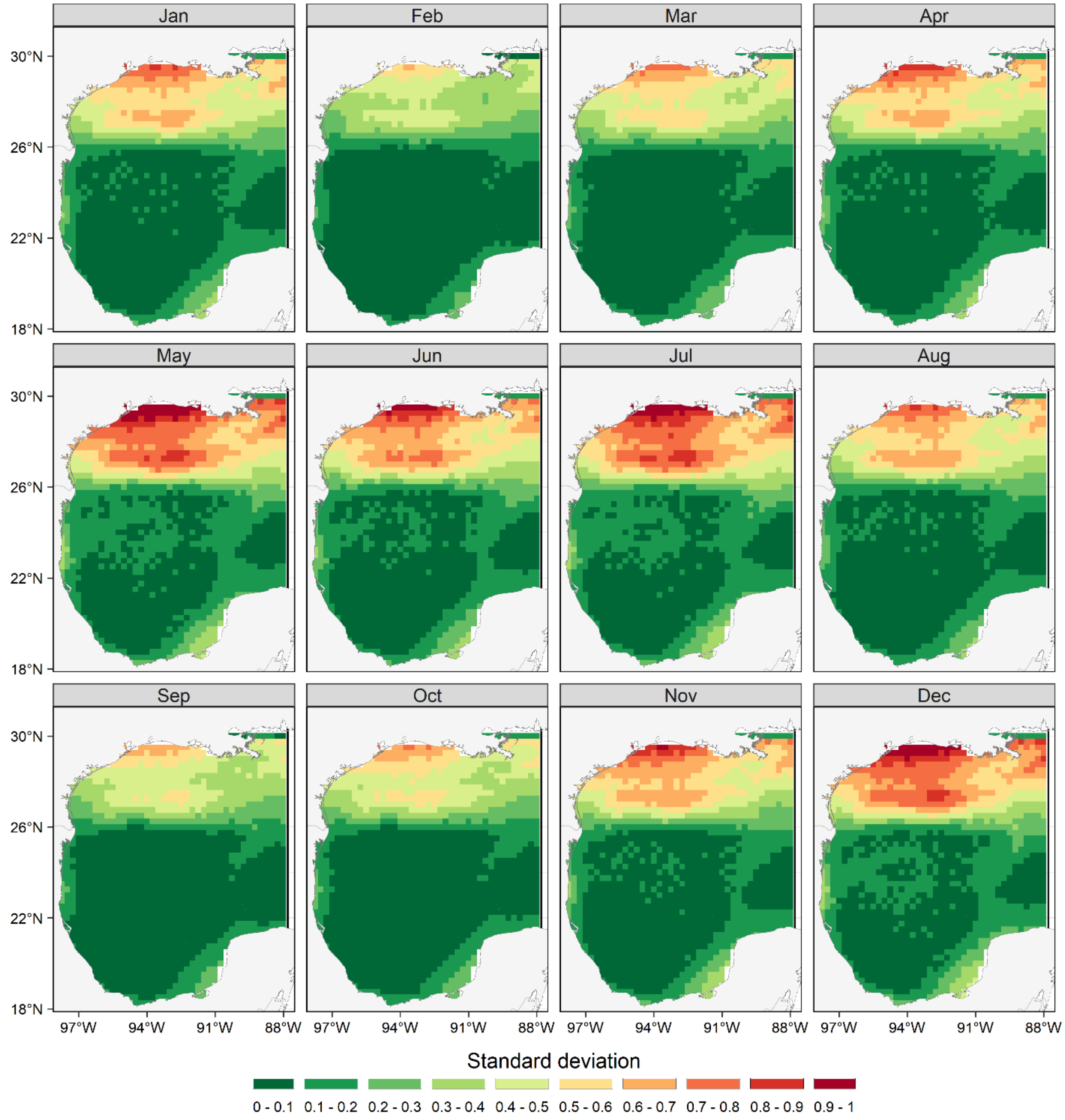

Fig. S11. Posterior median of the standard deviation of yellowfin tuna habitat suitability predictions. The maps were created with R's package "ggplot2" (https://ggplot2.tidyverse.org/), using the coastlines from the Global Self-consistent, Hierarchical, High-resolution Geography Database (http://www.soest.hawaii.edu/pwessel/gshhg/).

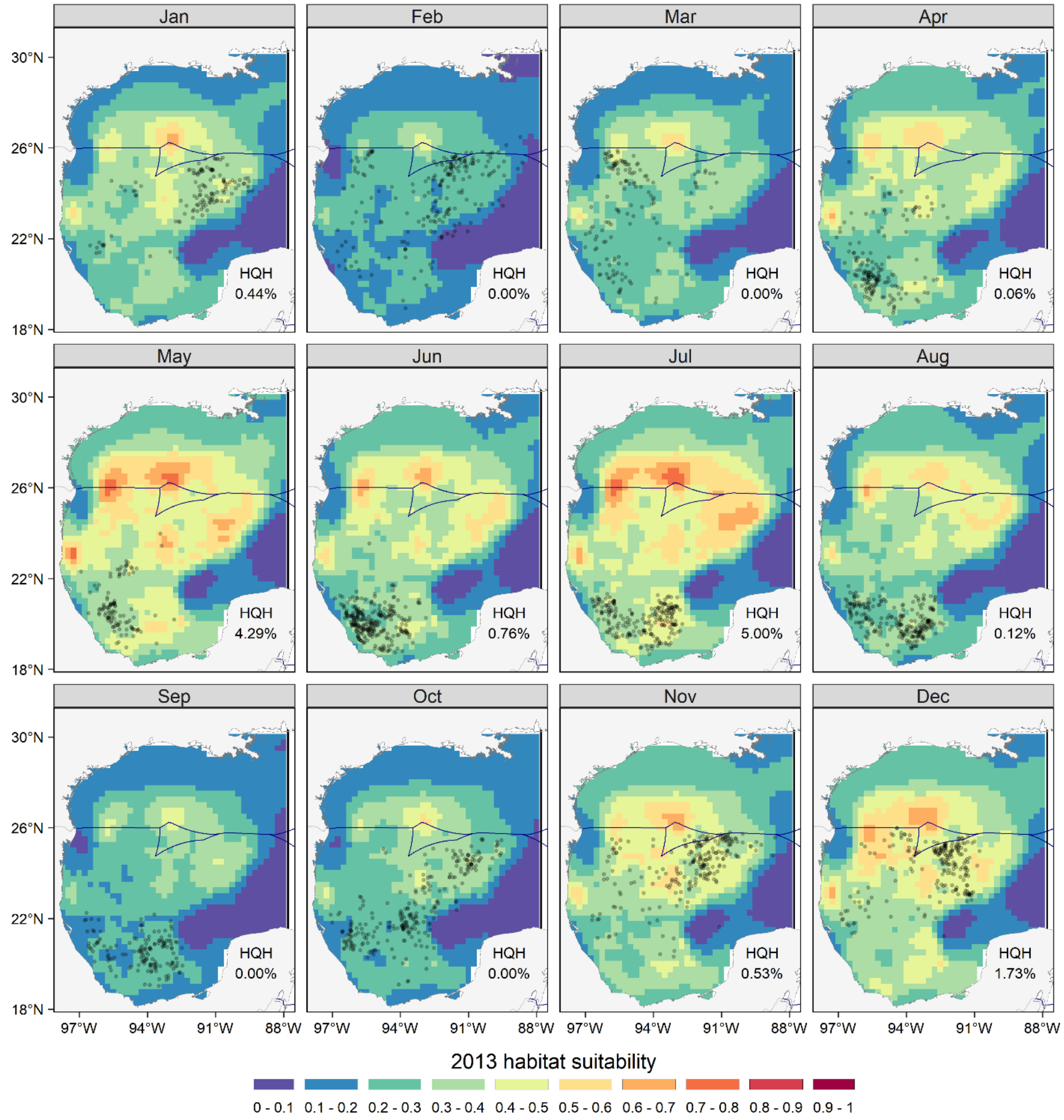

Fig. S12. Median predictions of yellowfin tuna habitat suitability for 2013 as an example of an average year at the interannual scale. The high quality (HQH) habitat percentage is the portion of the Gulf of Mexico with habitat suitability > 0.6. The blue line indicates the boundary between Mexican and U.S. Exclusive Economic Zones. Floating dots are the locations of longline sets (black dots). The maps were created with R's package "ggplot2" (https://ggplot2.tidyverse.org/), using the coastlines and political boundaries from the Global Self-consistent, Hierarchical, High-resolution Geography Database (http://www.soest.hawaii.edu/pwessel/gshhg/).

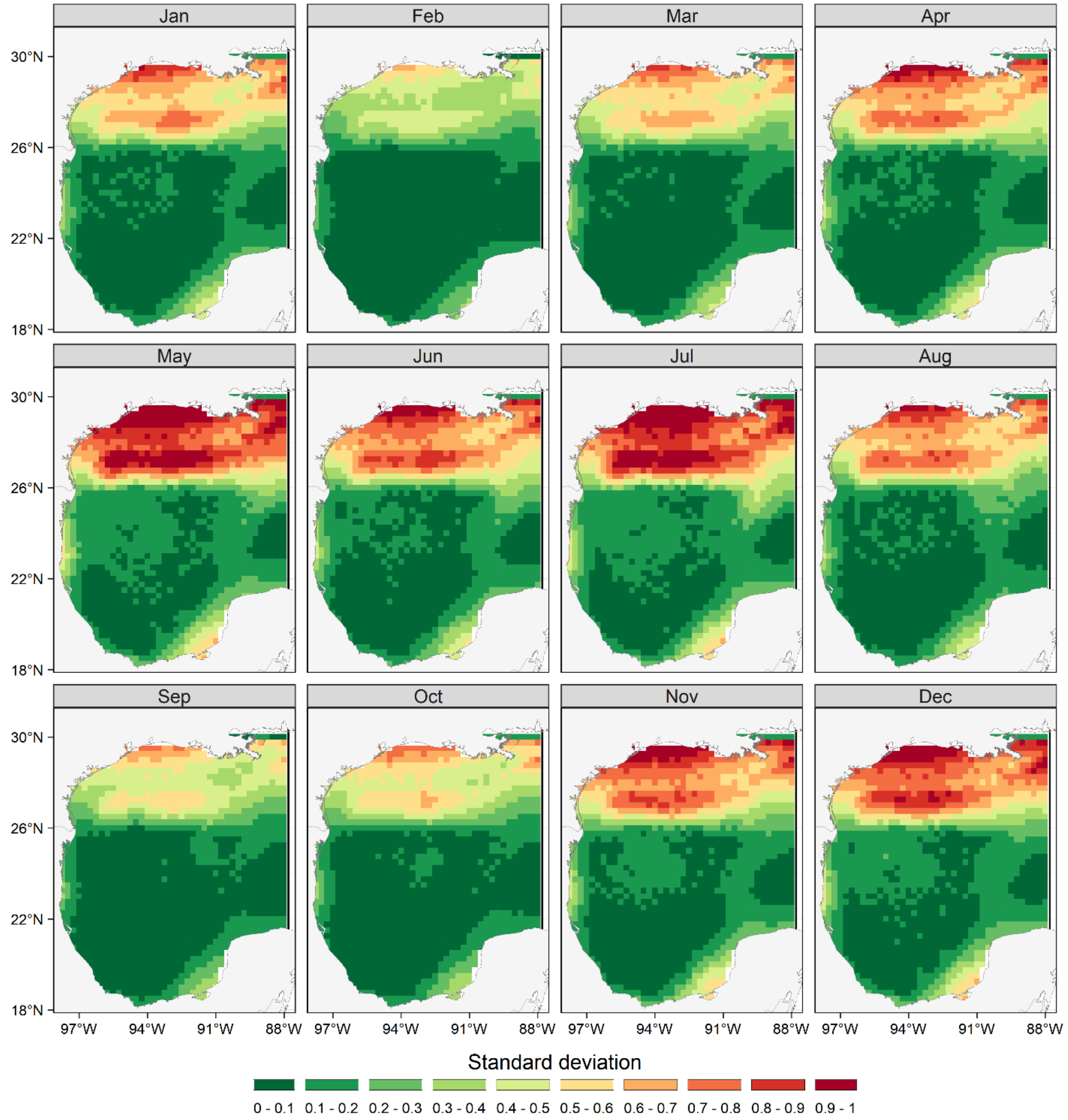

Fig. S13. Posterior standard deviation of the yellowfin tuna habitat suitability predictions for 2013 as an example of an average year at the interannual scale. The maps were created with R's package "ggplot2" (https://ggplot2.tidyverse.org/), using the coastlines from the Global Self-consistent, Hierarchical, High-resolution Geography Database (http://www.soest.hawaii.edu/pwessel/gshhg/).

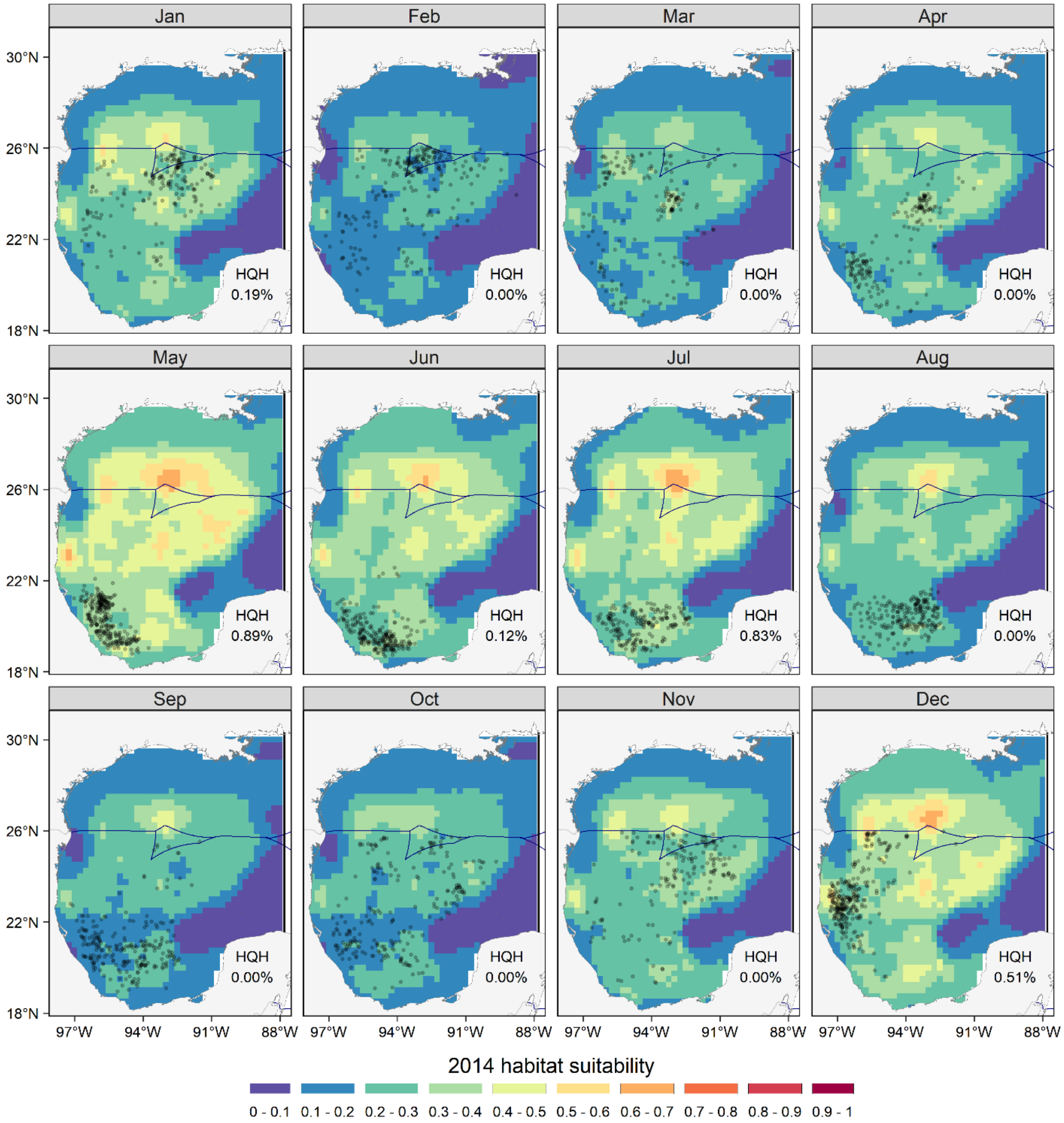

Fig. S14. Median predictions of yellowfin tuna habitat suitability for 2014 as an example of a negative year at the interannual scale. The high quality (HQH) habitat percentage is the portion of the Gulf of Mexico with habitat suitability > 0.6. The blue line indicates the boundary between Mexican and U.S. Exclusive Economic Zones. Floating dots are the locations of longline sets (black dots). The maps were created with R's package "ggplot2" (https://ggplot2.tidyverse.org/), using the coastlines and political boundaries from the Global Self-consistent, Hierarchical, High-resolution Geography Database (http://www.soest.hawaii.edu/pwessel/gshhg/).

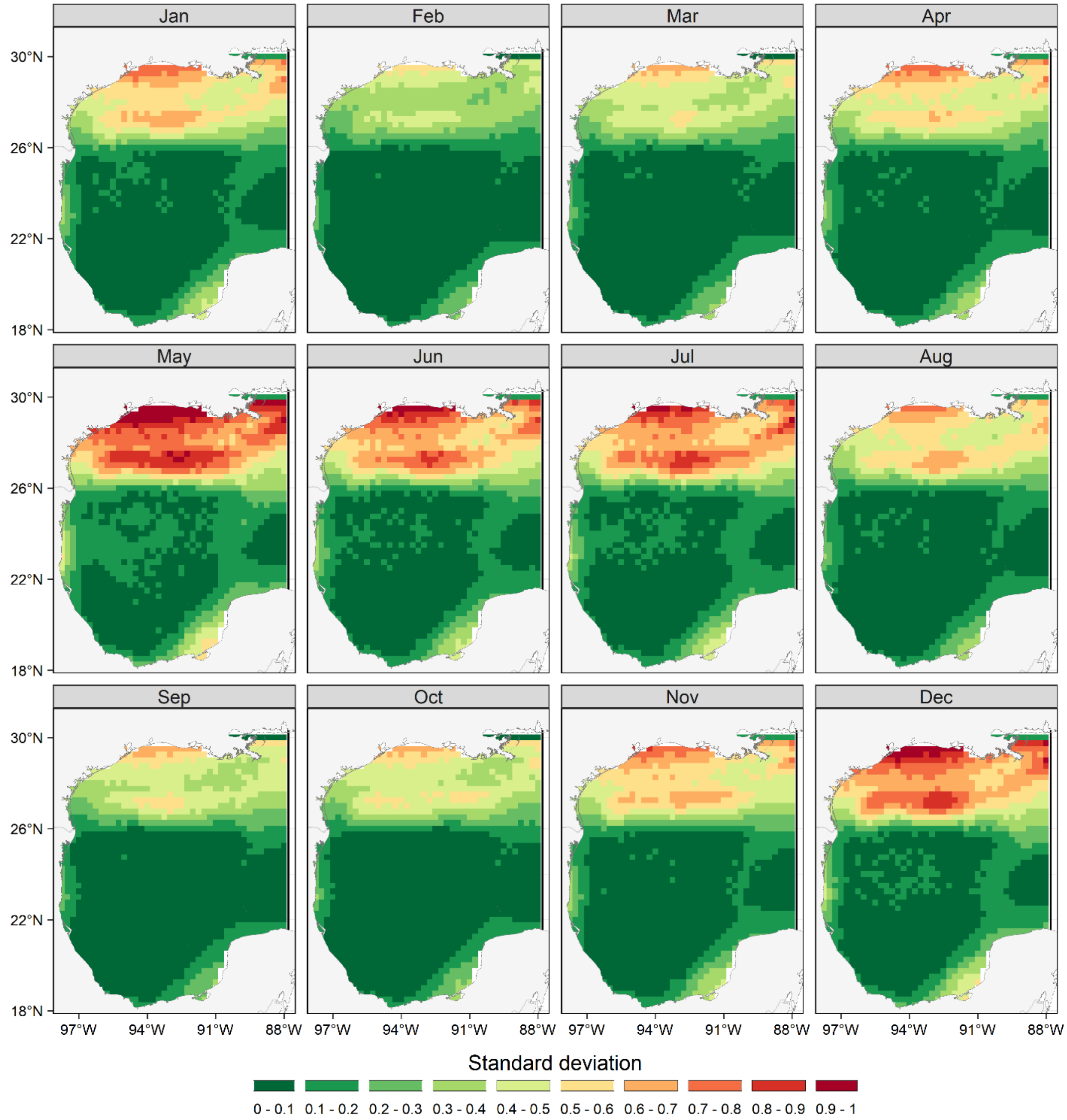

Fig. S15. Posterior standard deviation of the yellowfin tuna habitat suitability predictions for 2014 as an example of a negative year at the interannual scale. The maps were created with R's package "ggplot2" (https://ggplot2.tidyverse.org/), using the coastlines from the Global Self-consistent, Hierarchical, High-resolution Geography Database (http://www.soest.hawaii.edu/pwessel/gshhg/).

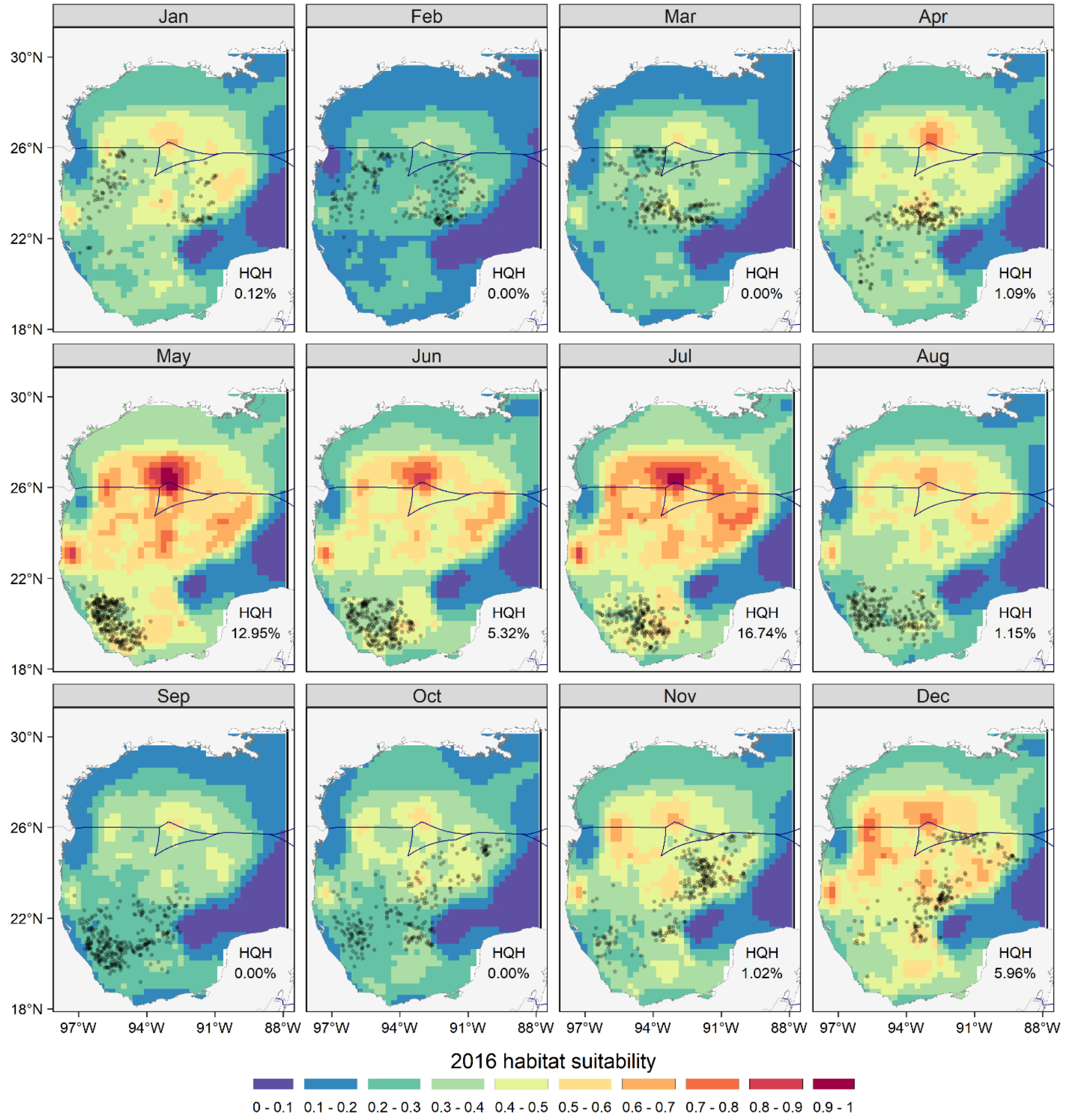

Fig. S16. Median predictions of yellowfin tuna habitat suitability for 2016 as an example of a positive year at the interannual scale. The high quality (HQH) habitat percentage is the portion of the Gulf of Mexico with habitat suitability > 0.6. The blue line indicates the boundary between Mexican and U.S. Exclusive Economic Zones. Floating dots are the locations of longline sets (black dots). The maps were created with R's package "ggplot2" (https://ggplot2.tidyverse.org/), using the coastlines and political boundaries from the Global Self-consistent, Hierarchical, High-resolution Geography Database (http://www.soest.hawaii.edu/pwessel/gshhg/).

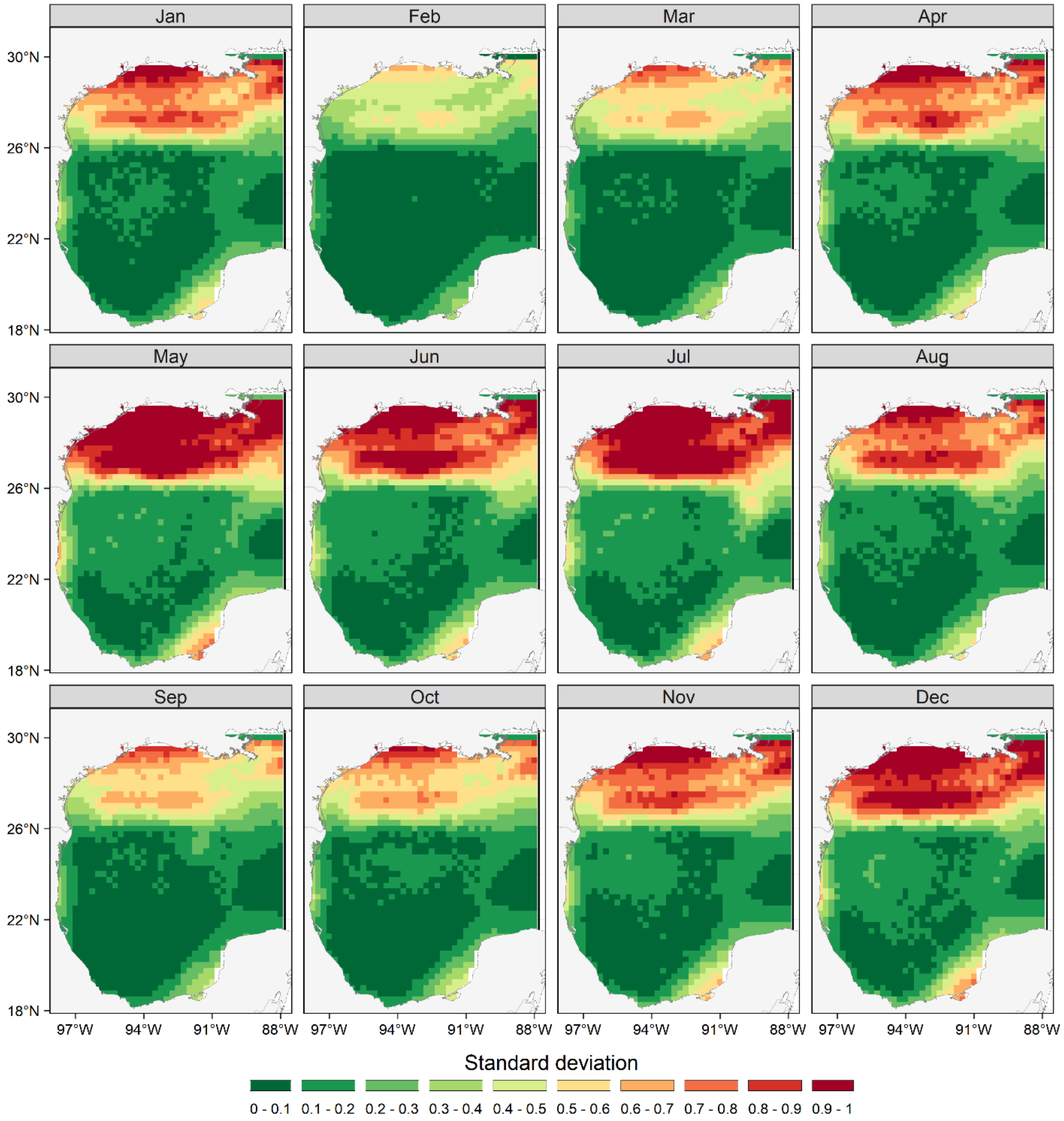

Fig. S17. Posterior standard deviation of the yellowfin tuna habitat suitability predictions for 2016 as an example of a positive year at the interannual scale. The maps were created with R's package "ggplot2" (https://ggplot2.tidyverse.org/), using the coastlines from the Global Self-consistent, Hierarchical, High-resolution Geography Database (http://www.soest.hawaii.edu/pwessel/gshhg/).

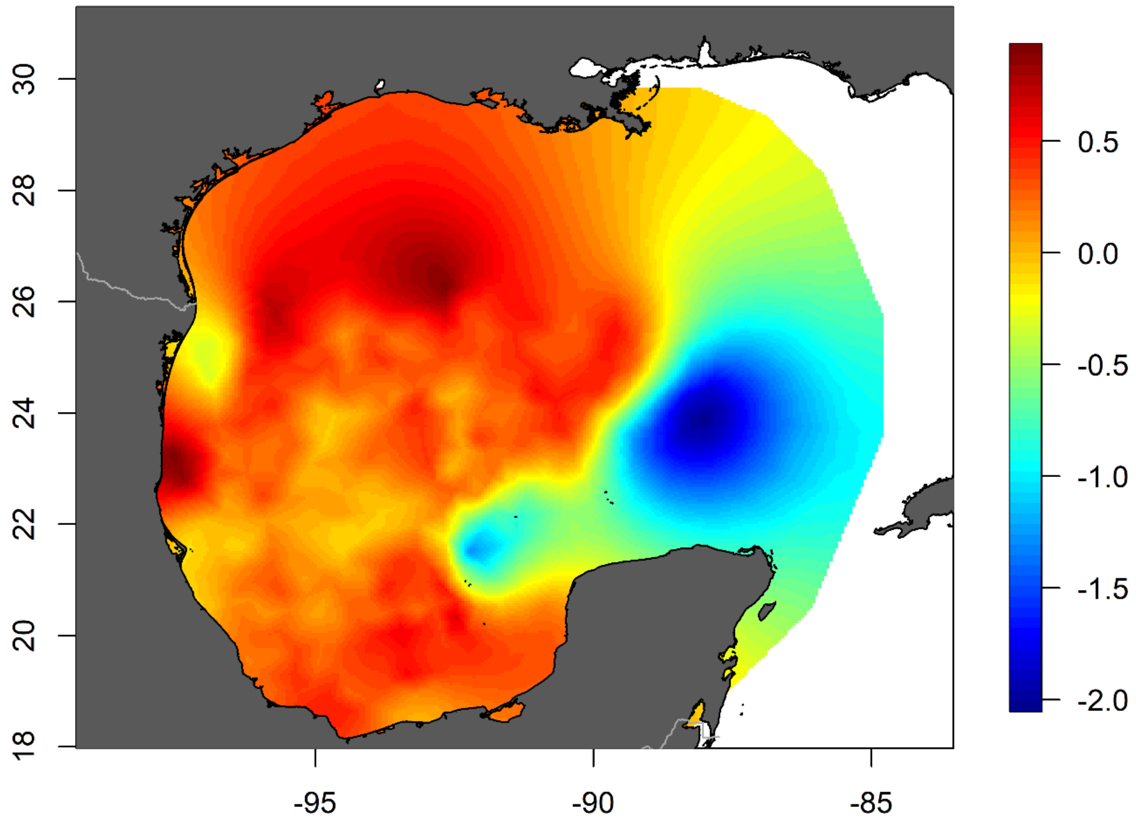

Fig. S18. Spatial random effect. The map was created with R's package "ggplot2" (https://ggplot2.tidyverse.org/ , using the coastlines and political boundaries from the Global Self-consistent, Hierarchical, High-resolution Geography Database (http://www.soest.hawaii.edu/pwessel/gshhg/).

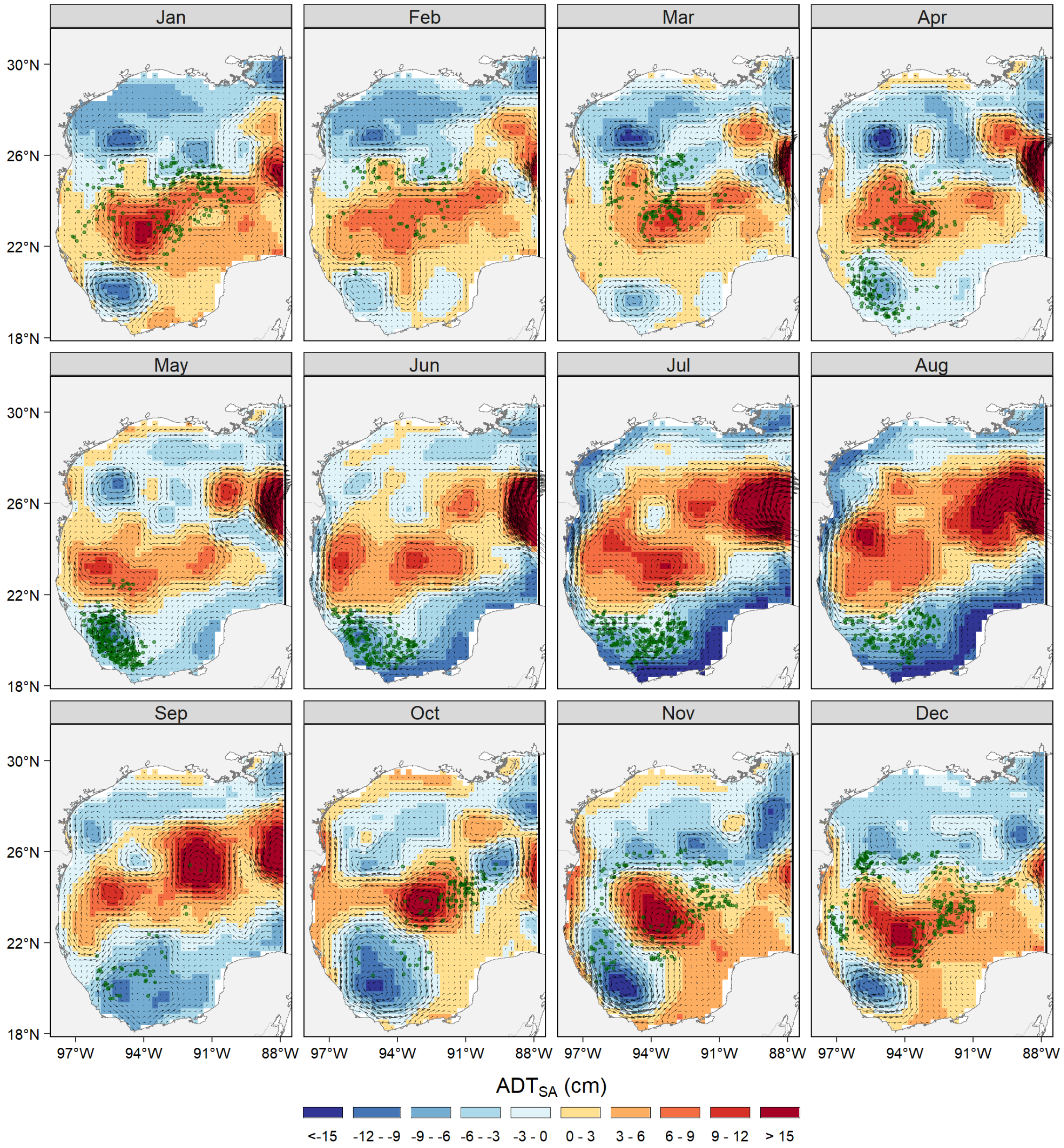

Fig. S19. Map of monthly averages (2012-2019) of absolute dynamic topography spatial anomalies (ADT_{SA}) used to model the climatological mean yellowfin tuna habitat suitability. Green dots indicate longline sets with a catch per unit effort greater than 30 individuals per 1000 hooks to facilitate visualization. The maps were created with R's package "ggplot2" (https://ggplot2.tidyverse.org/), using the coastlines and political boundaries from the Global Self-consistent, Hierarchical, High-resolution Geography Database (http://www.soest.hawaii.edu/pwessel/gshhg/).
